# Inhibition of autoantigen-induced B-cell receptor (BCR) internalization as a therapeutic strategy in diffuse large B cell lymphoma (DLBCL)

**DOI:** 10.1038/s41419-026-08446-1

**Published:** 2026-02-11

**Authors:** Patryk Górniak, Anna Polak, Anna Rams, Kristyna Kupcova, Eliza Głodkowska-Mrówka, Zofia Pilch, Marta Miączyńska, Dominika Nowis, Jakub Gołąb, R. Eric Davis, Ondrej Havranek, Przemysław Juszczyński

**Affiliations:** 1 Institute of Hematology and Transfusion Medicine, Warsaw, Poland; 2 BIOCEV, First Faculty of Medicine, Charles University, Prague, Czechia; 3 First Department of Medicine – Hematology, First Faculty of Medicine, Charles University and General University Hospital, Prague, Czechia; 4 Department of Immunology, Medical University of Warsaw, Warsaw, Poland; 5 International Institute of Molecular and Cell Biology, Warsaw, Poland; 6 Laboratory of Experimental Medicine, Medical University of Warsaw, Warsaw, Poland; 7 Department of Lymphoma and Myeloma, The University of Texas MD Anderson Cancer Center, Houston, TX USA

**Keywords:** B-cell lymphoma, Targeted therapies, Translational research

## Abstract

BCR signal dependency is a hallmark of diffuse large B-cell lymphoma (DLBCL) and other B-cell lymphoid malignancies originating from germinal centers. Chronic-active BCR signaling, typical for the more aggressive activated B-cell subtype (ABC) of DLBCLs, is often attributed to activating mutations within the BCR signaling cascade and continuous stimulation of the BCR by autoantigens. In certain ABC-DLBCLs, the BCR forms an intracellular multiprotein supercomplex with TLR9 and MYD88, which generates signals from endolysosomes. However, it is not clear whether the internalization of BCR is required for sustained signaling, nor have the mechanisms responsible for BCR trafficking been defined. To address these questions, we developed DLBCL cell models with modified ovalbumin (OVA)-specific hypervariable regions (HVRs) in the BCRs using CRISPR-Cas9 technology. Modified BCRs were incapable of binding self-antigens, while still responding in a controlled fashion to stimulation with ovalbumin. Using these genetic models, we demonstrated that autoantigens drive a complex BCR-dependent signaling program and facilitate the assembly of the intracellular BCR-TLR9-IκB complex, promoting NFκB pathway activation. Furthermore, we showed that the binding of autoantigens to the BCR leads to the internalization of the BCR–autoantigen complex via clathrin-mediated endocytosis (CME). Using genetic models with inducible inhibition of this endocytic pathway, we found that BCR internalization is essential for the oncogenic activation of the BCR-dependent signaling pathways and the formation of the BCR-TLR9-IκB complex in autoantigen-dependent ABC-DLBCL cells. Finally, CME inhibition with dynamin-2 antagonists, such as phenothiazine derivatives, reduces BCR signaling, cell viability, and synergizes with SYK and PI3Kδ inhibitors. Since phenothiazines have well-defined safety and pharmacokinetic profiles, our data provide a framework for the rational design of clinical trials employing these drugs in the autoantigen-dependent subset of DLBCL.

## Introduction

Diffuse large B-cell lymphoma (DLBCL) is the most common aggressive lymphoid malignancy in adults, accounting for up to 35% of non-Hodgkin lymphomas [1, 2]. DLBCLs are subdivided into the more aggressive, activated B-cell (ABC)-type, and the less aggressive, germinal center B-cell (GCB)-type [3]. While this classification provides fundamental insights into the molecular characteristics of DLBCL and has prognostic value, recent genomic profiling studies have revealed a more nuanced substructure within DLBCL, identifying at least five distinct subtypes with different mutational spectra [1, 4]. Despite well-characterized genetic heterogeneity, first-line DLBCL therapy lacks molecular personalization and is currently based on chemoimmunotherapy (R-CHOP or Pola-R-CHP) [5, 6]. Although approximately 60% of patients can be cured with this approach, the remaining 40% are refractory or relapse after initial response [5, 6]. Based on molecular characterization, numerous strategies were suggested and evaluated as potential strategies to improve R-CHOP efficacy in preclinical models or clinical trials (including lenalidomide, BTK, HDAC, PI3K, BCL2, PIM, and HSF1 inhibitors) [7–11]. Collectively, these studies call for comprehensive mechanistic analyses that connect genetic background with dynamic pathway regulation.

Although transformed B cells acquire new pro-survival mutations during the GC reaction, they frequently remain dependent on B-cell receptor (BCR) signaling [12, 13]. Similar to their normal counterparts, DLBCL cells exhibit two different patterns of signaling emanating from the BCR: tonic and chronic-active, characteristic of GCB and ABC subtypes, respectively [12, 13]. Tonic BCR signaling is antigen-independent, with phosphatidylinositide 3-kinase (PI3K) and AKT being the key mediators [12, 14–16]. In contrast, chronic-active BCR signaling engages multiple pathways and transcriptional networks, with the crucial role of NFκB activation. This mode of signaling resembles antigen-dependent BCR activation in normal B cells [13, 17]. While activating mutations in *CARD11* can explain the constitutive BCR signal in a fraction of cases, other mutations (e.g., in *CD79A/B* or *MYD88*, present in ~20-25% and up to 40% of ABC-DLBCLs, respectively) cannot initiate the signal by themselves [2, 13, 18]. Consistent with this, certain lymphomas exhibit non-random IGHV segment usage, suggesting antigen-dependent selection and signaling [19, 20]. In line with these findings, BCR engagement by autoantigens or homotypic BCR interactions has been identified in selected DLBCL cases [19–22].

Consistent with the pro-survival role of activated BCR signaling, targeting intracellular BCR-associated pathways with small-molecule inhibitors emerged as a new, promising therapeutic strategy. Subsequent studies clearly demonstrated that certain subgroups of DLBCL patients can benefit from this strategy, paving the way to further studies on strategies augmenting the activity of the BCR signal inhibitors [18, 23–26].

While the pro-survival role of autoantigens in DLBCL and other lymphomas is well-established, the proximal events triggering signaling pathways induced by BCR engagement are less well-defined. In certain ABC-DLBCLs, the BCR forms an intracellular multiprotein supercomplex with MYD88 and TLR9, generating signals from endolysosomes [27, 28]. However, it is not clear whether the internalization of the BCR is required for sustained signaling in DLBCL cells, nor have the mechanisms responsible for BCR trafficking been defined. Likewise, the role of autoantigens in this process remains undefined. To fill this gap, we generated DLBCL cell models with ovalbumin (OVA)-recognizing BCR hypervariable regions (HVRs). Cells with modified BCRs can no longer bind their original target autoantigens, but are responsive to stimulation with OVA under strictly controlled conditions. Our results show that BCR (auto)antigen engagement in DLBCL cells leads to the internalization of the BCR–antigen complex. This process is crucial for the formation of a previously reported signalosome complex containing the BCR, TLR9, and phosphorylated IκB [28], which triggers the canonical NFκB pathway. Furthermore, we demonstrate that disruption of clathrin-mediated endocytosis (CME) using dominant-negative dynamin-2 mutants decreases BCR internalization and DLBCL cell viability. Importantly, phenothiazine derivatives, acting as pharmacologic dynamin-2 antagonists, recapitulate these effects both in vitro and in vivo DLBCL xenograft models.

## Results

### Autoantigen binding is required for sustained BCR signaling in ABC-DLBCL cells

**Fig. 1:**
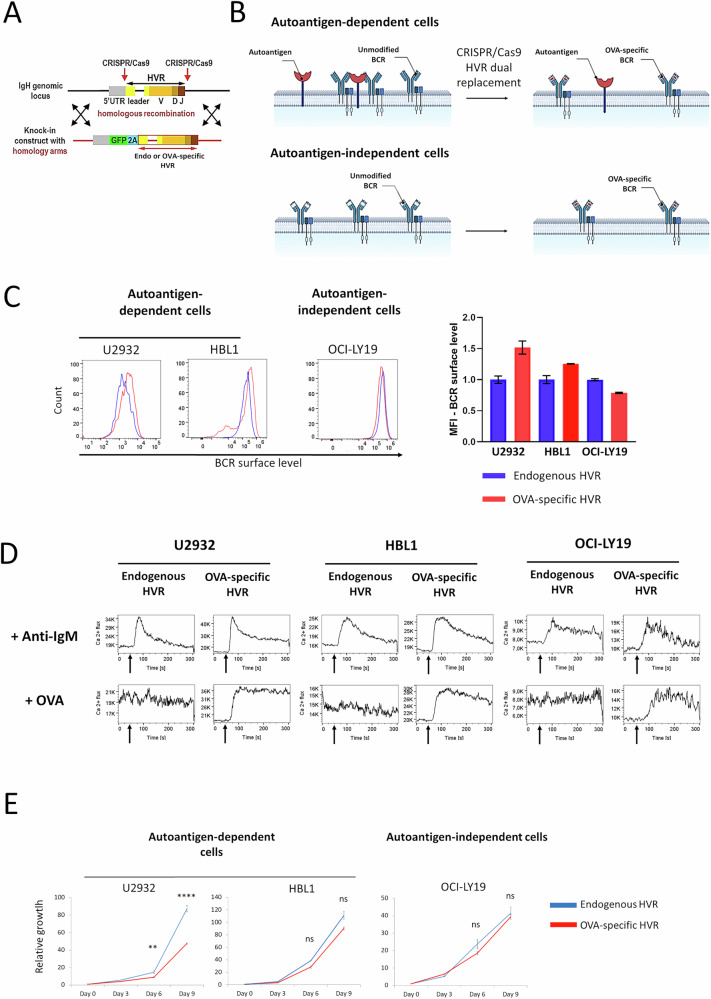
Strategy for modifying the hypervariable region (HVR) to alter BCR specificity and validation of cellular genetic models. **A** Schematic representation of the experimental strategy to replace the immunoglobulin heavy (IgH) and light (IgL) chain HVR fragments in DLBCL cells. Complementary DNA encoding a fluorescent protein (GFP for IgH, and the CFP variant mTurquoise2 for IgL) is followed by sequences encoding a 2A peptide, which creates a break during translation, a signal peptide, and either an OVA-specific HVR or an endogenous HVR. This construct was knocked into the start of the IgH and IgL translation sites using CRISPR-Cas9 methodology. **B** Scheme illustrating the predicted consequences of HVR replacement, leading to changes in BCR specificity and capabilities of autoantigen binding in autoantigen-dependent (ABC-type) and autoantigen-independent (GCB-type) cells. **C** Surface BCR levels in DLBCL cells with OVA-recognizing and endogenous HVRs, assessed by flow cytometry. Results are shown as histograms and median fluorescence intensity (MFI) plots (median ± standard deviation, SD) from two independent biological replicates. **D** Calcium fluxes in response to BCR stimulation in DLBCL cell models. Cells were incubated with anti-IgM (6 μg/mL) or OVA (1 μM). Arrows indicate the moments of OVA or anti-IgM addition. Experiments were performed in two or three independent biological replicates, and a representative experiment is shown. **E** Growth curves of U2932, HBL1, and LY19 cell lines with HVRs replaced to OVA-specific, compared to endogenous HVRs. Mean values from three independent biological replicates ± SD. *P* values were calculated using a two-sided t-test.

To investigate the proximal mechanisms by which autoantigen engagement sustains BCR signaling in DLBCL, we used CRISPR/Cas9 knock-in technology to modify BCR in lymphoma cell lines differing in their dependence on BCR–autoantigen interactions. Three ABC-type lines (TMD8, U2932, and HBL1) previously shown to rely on self-antigen engagement for survival [19] and an autoantigen-independent GCB-type line (OCI-LY19) were selected for modification. We replaced the hypervariable regions (HVRs) in the immunoglobulin heavy (IgH) and light chains (IgL) with ovalbumin (OVA)-recognizing HVRs derived from isolated OVA-reactive B cells [29]. With this approach, cells with fully modified HVR express GFP (IgH-derived) and mTurquoise (IgL-derived) and can be isolated using FACS (Supplementary Fig. 1). Endogenous HVRs from the cell lines being targeted were used to generate appropriate controls (Fig. 1A).

While attempts to introduce OVA-specific BCRs in TMD8 cells were unsuccessful (likely due to their strict dependence on autoantigen-induced BCR signaling) [19], successful modification was achieved in U2932 and HBL1 cells. The GCB-type OCI-LY19 line, used as a control, was efficiently modified and expressed OVA-specific BCRs as expected. Collectively, this approach successfully produced DLBCL cell models with OVA-recognizing BCRs that no longer bind cognate autoantigens (Fig. 1B), enabling studies on the mechanisms of autoantigen-induced signal generation in DLBCL cells.

In cell lines that naturally express autoantigen-specific BCRs, replacement with OVA-reactive HVRs caused an increase in cell-surface BCR levels. In contrast, OVA-reactive BCR surface levels in the OCI-LY19 cells were not increased when compared to the control cells (Fig. 1C). Both the native and the dual HVR-replaced BCRs were capable of signal generation, as demonstrated by calcium flux analysis following anti-IgM-mediated receptor cross-linking (Fig. 1D). However, OVA stimulation induced calcium fluxes only in cells with OVA-reactive HVRs (Fig. 1D), confirming the functionality and antigen specificity of the engineered BCRs.

As expected, OCI-LY19 (GCB-DLBCL) growth rates were similar in cells with endogenous or OVA-specific BCRs, indicating antigen-independent signaling. In contrast, U2932 (ABC-DLBCL) cells showed reduced proliferation following HVR replacement, despite elevated surface BCR expression. HBL1 cells displayed only a minor reduction, suggesting partial signaling compensation (Fig. 1E). Collectively, these findings confirm the dependence of ABC-DLBCL cells on autoantigen-driven BCR activation while indicating their capacity to engage compensatory signaling pathways when antigen binding is impaired.

**Fig. 2:**
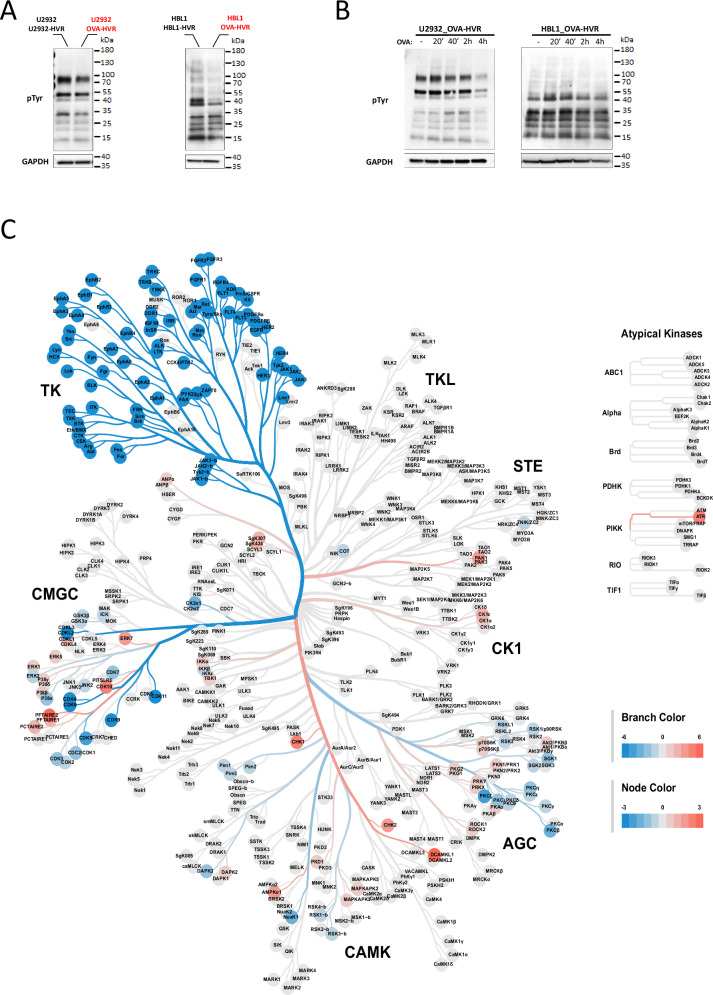
Autoantigen binding activates oncogenic BCR signaling in DLBCL cells. **A** Western blot analysis of tyrosine-phosphorylated proteins in cells with OVA-recognizing and endogenous HVRs. GAPDH was used as a loading control. The experiment was performed in two independent biological replicates, and a representative blot is shown in the figure. Uncropped immunoblot images are provided in the Supplementary Material. **B** Western blot analysis of tyrosine-phosphorylated proteins in cells with OVA-recognizing HVRs following stimulation with OVA (1 μM) for the indicated times. GAPDH was used as a loading control. Uncropped immunoblot image is provided in the Supplementary Material. **C** Differential analysis of kinase activity levels in HBL1 cells with OVA-specific HVRs compared to those with endogenous HVRs, presented as a kinome tree (values>0 indicate higher activity in OVA-reactive cells). Each cell model was evaluated in two independent biological replicates, data represent the average values. AGC – *Protein Kinase A, G, and C family;* CAMK – *Calcium/Calmodulin-dependent Protein Kinase;* CK1 – *Casein Kinase 1 family;* CMGC – *CDK, MAPK, GSK3, and CLK kinases;* TK – *Tyrosine Kinases and* TKL – *Tyrosine Kinase-Like*.

To characterize the autoantigen-dependent signaling, we first assessed tyrosine-phosphorylated proteins in ABC-DLBCL cells with OVA-specific and endogenous HVRs. In both HBL1 and U2932, cells expressing the OVA-specific BCR exhibited decreased tyrosine phosphorylation of numerous proteins when compared to controls (Fig. 2A and Supplementary Fig. 2). Incubation with OVA upregulated tyrosine phosphorylation in these proteins (Fig. 2B). Next, we compared global tyrosine and serine/threonine kinase activities in OVA-specific and control cells using the chip-based phosphoproteomic PamGene platform. This functional kinase assay measures protein kinase activity directly in cellular lysates by quantifying phosphorylation of peptides printed on a chip (Supplementary Fig. 3). HBL1 cells with OVA-specific BCRs (incapable of autoantigen binding) exhibited significantly reduced activity of proximal BCR signaling mediators, including SRC family kinases (e.g., LYN, FYN, BLK), SYK tyrosine kinase, Bruton’s tyrosine kinase (BTK), and protein kinase C. Furthermore, these OVA-specific cells also showed decreased activity of kinases essential for the regulation of cell proliferation (cyclin-dependent kinases, CDKs), protein biosynthesis (ribosomal S6 kinases, RSKs), and kinases engaged in oncogenic signaling (JAKs, PIMs, FLT1/3/4, PDGFRs, FGFRs, TRKA/B/C) (Fig. 2C and Supplementary File 1).

**Fig. 3:**
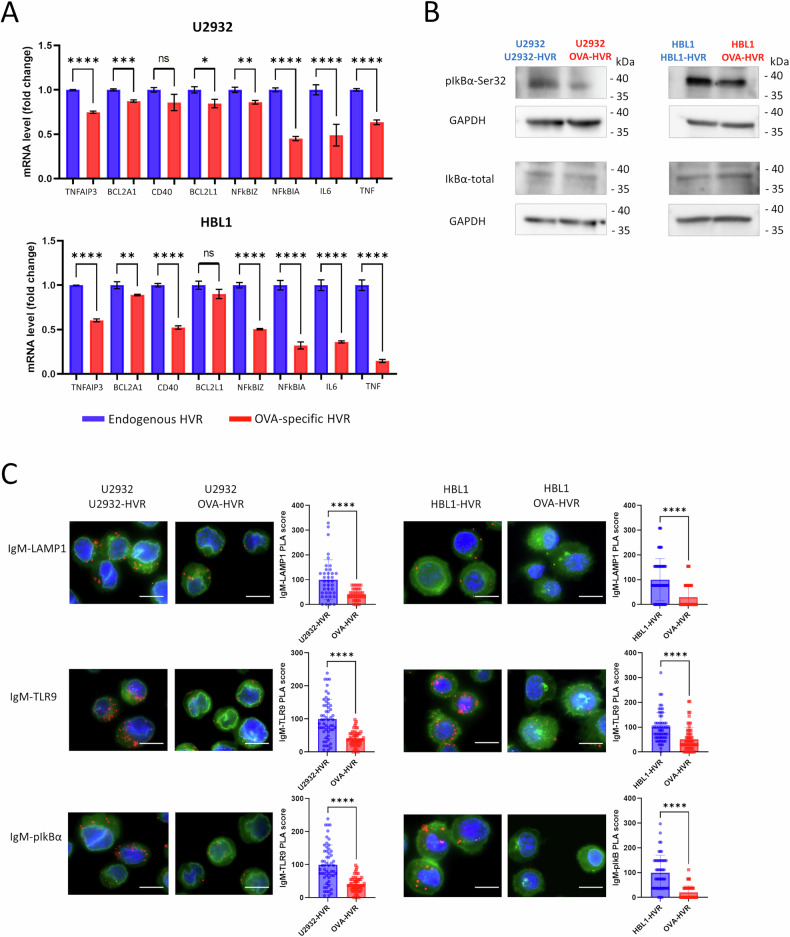
Autoantigen binding induces the assembly of the BCR-TLR9 complex in the endosomal compartment and activates NFκB pathway in DLBCL cells. **A** NFκB-dependent gene expression in cells with OVA-recognizing and endogenous HVRs. Relative mRNA expression was assessed by real-time PCR using GAPDH as a reference gene. Data from three independent replicates are shown, with error bars representing SD. *P* values were calculated by using two-sided unpaired t*-*test. **B** Western blot analysis of phospho-IκBα in U2932 and HBL1 cells with OVA-recognizing and endogenous HVRs. GAPDH was used as a loading control. Experiments were performed in two independent biological replicates, and a representative blot is shown. Uncropped immunoblot image is provided in the Supplementary Material. **C** Proximity ligation assays showing co-localization (red puncta) of IgM-LAMP1, IgM-TLR9, and IgM-pIκBα in genetic models with OVA-recognizing and endogenous HVRs. Nuclei were stained with DAPI (blue), and membranes were visualized using WGA (green). To determine the Proximity Ligation Assay (PLA) score, the number of PLA puncta per cell was quantified in three independent OVA-specific samples and three control samples. Values from OVA-specific cells were normalized to the mean number of puncta in control cells (set to 100). Each dot in the graph represents the normalized number of PLA puncta detected in a single cell. Box-and-whisker plots display the median PLA score, with whiskers incorporating 10–90% of all data. *P* values were determined using a two-sided t-test; ****P ≤ 0.0001. Scale bar, 10 μm.

Since altering BCR specificity and eliminating autoantigen binding decreased the activity of multiple BCR-related kinases and global tyrosine phosphorylation, we evaluated the changes in NFκB activity in engineered cells. For this purpose, we evaluated the phosphorylation levels of the NFκB inhibitory protein IκBα and the expression of NFκB-dependent genes as markers of NFκB activity. These analyses revealed significantly higher levels of IκBα phosphorylation and NFκB-dependent transcripts in the autoantigen-reactive cells, highlighting the crucial role of sustained BCR–autoantigen engagement for continuous NFκB activation (Fig. 3A, B).

In a subset of ABC-DLBCLs (referred to as MCD or C5 clusters) [1, 4], NFκB pathway activation is mediated by the intracellular BCR-TLR9 complex [28]. Using Proximity Ligation Assay (PLA) analysis, we found that in OVA-reactive cells, the assembly of the endolysosomal BCR-TLR9 complex (as indicated by interaction with the LAMP1 marker) was significantly inhibited. Furthermore, the interaction between this complex and the phosphorylated form of IκBα, which is critical for NFκB activation, was also diminished in OVA-reactive models (Fig. 3C).

Collectively, these findings suggest that autoantigen binding by the BCR in ABC-DLBCL cells facilitates the recruitment of BCR and TLR9 into a functional endolysosomal BCR-TLR9 complex, contributing to NFκB activation.

### Antigen stimulation in DLBCL cells leads to BCR internalization

DLBCL cells with OVA-specific BCRs exhibited increased BCR surface density and reduced formation of the endolysosomal BCR-TLR9 complex (Figs. 1C and 3C), indicating that (auto)antigens induce BCR internalization, and—consequently—loss of autoantigenic stimulation results in BCR membrane retention.

**Fig. 4:**
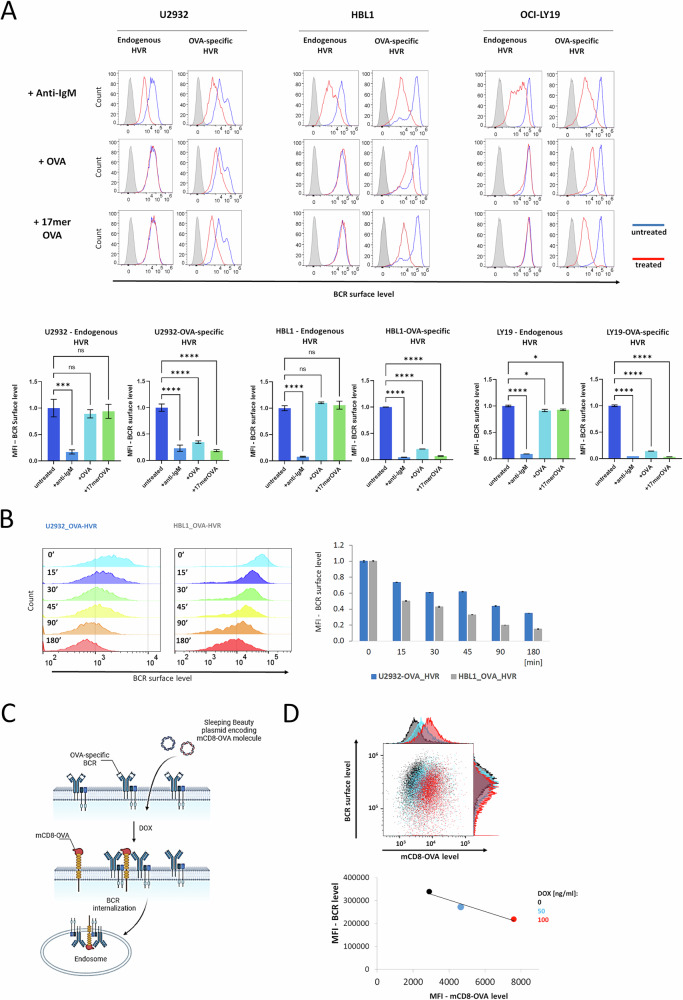
Antigenic stimulation in DLBCL cells leads to the BCR internalization. **A** BCR internalization is observed as a decrease in surface BCR levels in response to BCR stimulation in DLBCL cell models. Cells were incubated with anti-IgM (6 μg/mL), OVA (1 μM), or an OVA-17mer-biotin complexed with avidin for 3 h. Surface BCR levels were assessed by flow cytometry. Results are shown as histograms and MFI plots (median ± SD) from three independent biological replicates. *P* values were calculated by using two-sided unpaired t-test. **B** Time course of surface BCR decrease in response to OVA treatment (1 μM) in cells with OVA-specific HVRs. Surface BCR levels were assessed by flow cytometry. Results are shown as histograms and MFI plots (median ± SD) from three independent replicates. **C** Schematic representation of BCR internalization in DLBCL genetic models, mimicking the co-expression of BCRs and autoantigens on the plasma membrane. The cell model was derived from the autoantigen-independent OCI-LY19 GCB-DLBCL cell line, showing stable expression of OVA-specific BCRs and doxycycline (Dox)-inducible expression of a transmembrane protein, a truncated murine CD8a fused to the cognate OVA peptide (mCD8-OVA). **D** Surface levels of OVA-specific BCR and mCD8-OVA in the OCI-LY19-based genetic model. Cells were treated with DOX (50 or 100 ng/mL) for 24 h and assessed by flow cytometry. The results shown are from representative experiment out of three independent biological replicates and are presented as dot plots, histograms, and MFI plots.

To directly confirm this scenario, we incubated OVA-specific cell models with anti-IgM (a positive control), OVA, and 17mer-OVA peptide conjugated with biotin and complexed with avidin. Anti-IgM triggered BCR endocytosis in both HVR types, but OVA or 17mer-OVA exhibited this effect only in cells with OVA-specific HVRs (Fig. 4A). Time-course analysis demonstrated that BCR internalization is a dynamic process, resulting in a significant decrease in BCR surface levels within minutes and progressing up to 3 h (Fig. 4B).

Since BCR-engaging DLBCL autoantigens can be either extracellular or expressed on the plasma membrane [19], we asked whether membrane-anchored autoantigens could also initiate BCR endocytosis. To address this question, we generated a model derived from the autoantigen-independent OCI-LY19 GCB-DLBCL cell line with OVA-specific BCRs. This cell line was modified to express a doxycycline (Dox)-inducible construct including the truncated murine CD8a (mCD8) fused to the OVA peptide (Fig. 4C). In this model, Dox-induced expression of membrane-anchored mCD8-OVA resulted in a significant decrease of the surface BCR levels, demonstrating that membrane-expressed autoantigens can initiate BCR internalization in DLBCL cells (Fig. 4D).

### Inhibition of BCR internalization blocks BCR-TLR9 complex formation and pro-survival signaling in ABC-DLBCL cells

Given the observations that altered BCR specificity and loss of autoantigen binding led to BCR membrane retention, decreased formation of intracellular BCR-TLR9 supercomplexes, and reduced NFκB activity, we hypothesized that blocking BCR internalization might represent a novel DLBCL therapeutic strategy.

To evaluate the consequences of inhibition of endocytosis for DLBCL cell survival, we first utilized available datasets from CRISPR-Cas9 screening studies [2] and the KEGG Endocytosis Human Gene Set. With this approach, we found that knockout of multiple genes in the clathrin-mediated endocytosis (CME) pathway resulted in reduced DLBCL cell viability (Supplementary Fig. 4). CME is responsible for the internalization of multiple surface receptors, including BCR–antigen complexes [30, 31]. DNM2 (dynamin-2) GTPase plays an essential role in CME. DNM2 forms ring-like structures around the neck of the invaginating vesicle and facilitates the scission of endocytic vesicles from the cell membrane. Importantly, DNM2 depletion exhibited a stronger effect on DLBCL survival than depletion of other genes coding for proteins targeted by available small-molecule inhibitors (Supplementary Fig. 5).

**Fig. 5:**
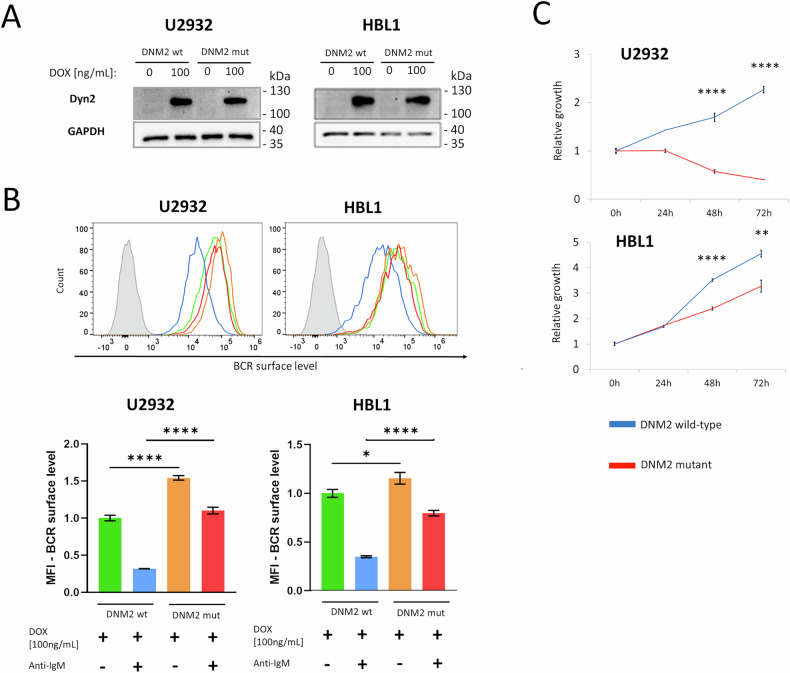
BCR internalization is dynamin-2-dependent and is crucial for sustaining the growth of DLBCL cells. **A** Western blot analysis showing the induction of expression of the wild-type Dynamin-2 (DNM2wt) and the dominant-negative K44A mutant (DNM2mut) in U2932- and HBL1-derived genetic models after treatment with doxycycline (DOX) (100 ng/mL for 24 h). GAPDH was used as a loading control. Two independent biological replicates were performed, and a representative blot is shown. Uncropped immunoblot images are provided in the Supplementary Material. **B** Surface levels of BCR in cells expressing DNM2wt or DNM2mut. Cells were pretreated with DOX (100 ng/mL) for 24 h and stimulated with anti-IgM (6 μg/mL) for 3 h. Flow cytometry analysis results are shown as histograms and MFI plots (median ± SD) from three independent biological replicates. *P* values were calculated by using two-sided unpaired t-test. The line colors on the histograms correspond to the bar colors on the MFI chart. **C** Absolute growth curves of U2932 and HBL1 cells expressing DNM2wt or DNM2mut. To induce DNM2wt/mut, cells were treated with DOX (100 ng/mL). Data represent mean of three independent biological replicates ± SD. *P* values were calculated using a two-sided t-test.

To assess the role of DNM2 and CME in autoantigen-induced BCR internalization and the subsequent activation of BCR signaling in DLBCL cells, we generated cellular models with inducible expression of a dynamin-2 K44A mutant lacking GTPase activity and shown to inhibit CME in a dominant-negative manner (DN-DNM2) (Fig. 5A) [32, 33]. Overexpression of DN-DNM2 markedly decreased internalization of the transferrin receptor, confirming the inhibition of CME (Supplementary Fig. 6). DN-DNM2 expression increased BCR surface levels in untreated, autoantigen-dependent cells. Likewise, DN-DNM2 blocked BCR internalization after anti-IgM cross-linking (Fig. 5B). Consistent with the data from the CRISPR-Cas9 screen, DN-DNM2 expression resulted in growth inhibition in the generated cell models, particularly in U2932 cells (Fig. 5C).

**Fig. 6:**
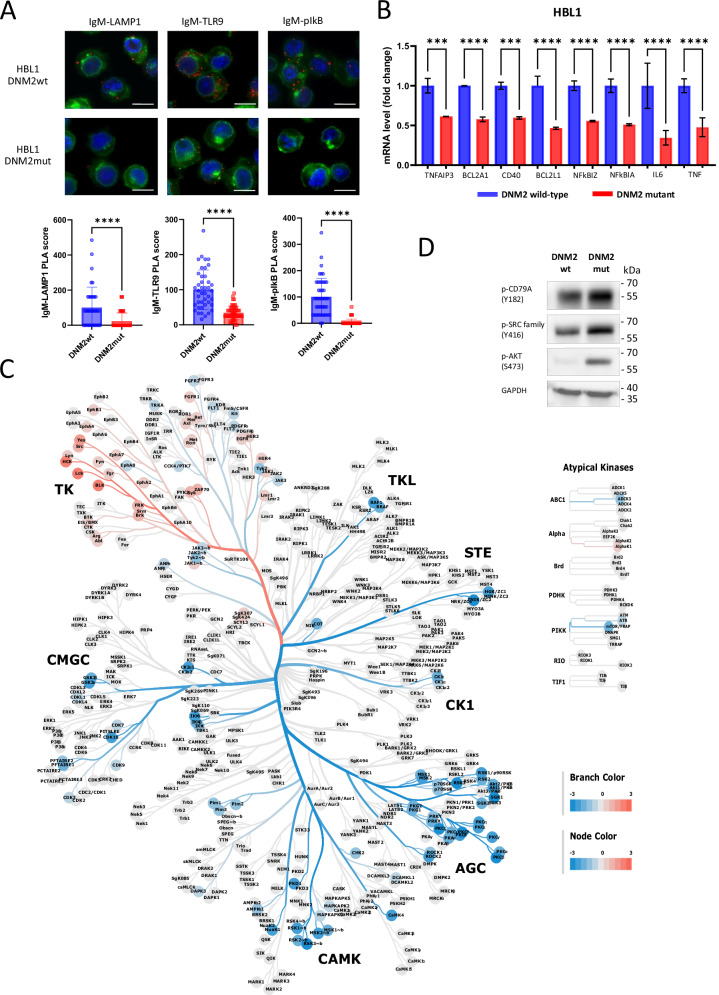
Inhibition of BCR internalization blocks BCR-TLR9 complex formation and pro-survival signaling in ABC-DLBCL cells. **A** Proximity ligation assays showing co-localization (red puncta) of IgM-LAMP1, IgM-TLR9, and IgM-pIκBα in HBL1 cells expressing DNM2wt or DNM2mut. Nuclei were stained with DAPI (blue), and membranes were visualized with WGA (green). To determine the Proximity Ligation Assay (PLA) score, the number of PLA puncta per cell was quantified in three independent DNM2mut samples and three DNM2wt samples. Values from DNM2mut cells were normalized to the mean number of puncta in control cells (set to 100). Each dot in the graph represents the normalized number of PLA puncta detected in a single cell. Box-and-whisker plots display the median PLA score, with whiskers incorporating 10–90% of all data. *P* values were determined using a two-sided t-test; ****P ≤ 0.0001. Scale bar, 10 μm. **B** Expression of NFκB-dependent genes in HBL1 cells expressing DNM2mut or DNM2wt. To induce DNM2wt/mut, cells were incubated with DOX (100 ng/mL) for 24 h. Relative mRNA expression was assessed by real-time PCR using GAPDH as a reference gene. Data from three independent biological replicates are shown, with error bars representing SD. *P* values were calculated by using two-sided unpaired t*-*test. **C** Differential analysis of kinase activity in HBL1 cells expressing DNM2mut versus DNM2wt, presented as a kinome tree (>0 indicates higher activity in OVA-reactive cells). To induce DNM2wt/mut, cells were incubated with DOX (100 ng/mL) for 24 h. Each cell model was evaluated in two independent biological replicates, data present the average values. **D** Western blot analysis of phosphorylated proximal BCR signaling mediators in HBL1 cells expressing DNM2mut and DNM2wt variants after treatment with doxycycline (DOX) (100 ng/mL for 24 h). The α-SRC antibody cross-reacts with LYN, FYN, LCK, YES, and HCK. GAPDH was used as a loading control. Experiments were performed in two independent biological replicates, and a representative blot is shown. Uncropped immunoblot image is provided in the Supplementary Material.

BCR membrane retention after CME inhibition suggested reduced endolysosomal BCR-TLR9 complex formation. Indeed, CME inhibition in HBL1 cells led to a marked decrease in BCR-TLR9-pIκB complexes (Fig. 6A), which subsequently resulted in the downregulation of NFκB-dependent gene expression (Fig. 6B).

To further characterize the consequences of CME inhibition, we evaluated the changes in tyrosine and serine-threonine kinase activity in HBL1 cells after DN-DNM2 induction using the PamGene platform. Inhibition of CME dampened the activation of several signaling pathways (mTOR, CDKs, PKCs, and RSKs), similar to the effects of switching endogenous HVRs for OVA-recognizing HVRs. However, unlike the genetic change in BCR specificity, CME inhibition led to increased activity of proximal BCR signaling kinases, including SRC family kinases (including LYN) and SYK—a critical tyrosine kinase involved in tonic BCR signaling [34]. Consistent with this, DNM2 inhibition increased the activity of SRC family kinases (including LYN) and AKT, the key kinase mediating tonic BCR signaling [12, 16] (Fig. 6C, D and Supplementary File 2). These observations suggest that blocking endocytosis increases surface BCR density, which may enhance tonic BCR signaling and represent a cellular attempt to compensate for the loss of the chronic-active signal.

### Phenothiazine derivatives inhibit antigen-induced BCR internalization

**Fig. 7:**
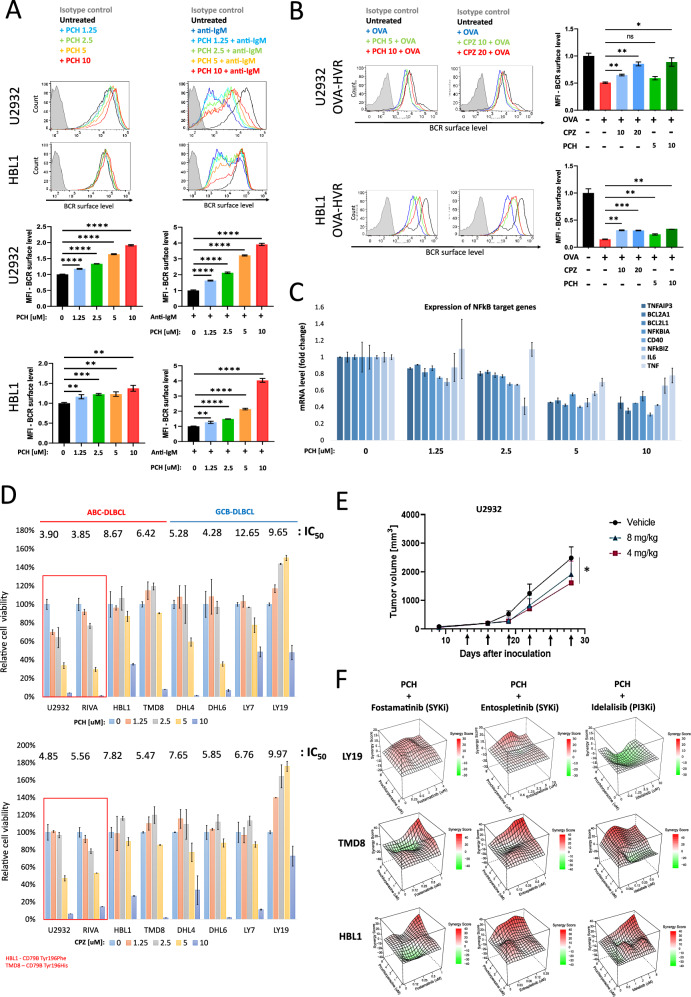
Phenothiazine derivatives inhibit autoantigen-induced BCR internalization and DLBCL cell growth. **A** BCR internalization in phenothiazine derivative-treated DLBCL cells. U2932 and HBL1 cells were treated with DMSO or the indicated concentrations of prochlorperazine (PCH) for 16 h and then stimulated with anti-IgM (6 μg/mL for 1 h) to induce BCR internalization. Surface BCR levels were assessed by flow cytometry. Results are presented as histograms and MFI plots (median ± SD) of three independent biological replicates. *P* values were calculated by using two-sided unpaired t-test. **B** BCR internalization in phenothiazine derivative-treated OVA-specific DLBCL cells. Genetic models expressing OVA-specific BCRs were treated with DMSO, prochlorperazine (PCH) (5 and 10 μM), or chlorpromazine (CPZ) (10 and 20 μM) for 16 h and then stimulated with OVA (1 μM for 1 h) to induce BCR internalization. Surface BCR levels were assessed by flow cytometry. Results are presented as histograms and MFI plots (median ± SD) of two independent biological replicates. *P* values were calculated by using two-sided unpaired t*-*test. **C** Expression of NFκB-dependent genes in U2932 treated with DMSO or the indicated concentrations of prochlorperazine (PCH) for 16 h. Relative mRNA expression was assessed by real-time PCR using GAPDH as a reference gene. Data from three independent replicates are shown, with error bars representing SD. *P* values were calculated by using two-sided unpaired t*-*test. **D** Toxicity of prochlorperazine (PCH) and chlorpromazine (CPZ) in DLBCL cell lines. Cells were incubated for 72 h with DMSO or the indicated concentrations of drugs. Afterward, cells were stained with SYTOX Red, and live cells were counted by flow cytometry. Graphs show mean values from three independent biological experiments, with error bars representing SD. **E** Tumor growth kinetics in prochlorperazine or vehicle-treated mice. U2932 cells were suspended in 30% Matrigel and injected into NSG mice. When tumor volume reached ≥100 mm³, mice were treated with intraperitoneal injections of prochlorperazine (4 mg/kg or 8 mg/kg) or vehicle (H₂O) as indicated. Each experimental group consisted of five mice. Asterisks indicate *P* values < 0.05 in a one-sided Student’s t-test. **F** Drug synergy in TMD8, HBL1, and OCI-LY19 cells treated with prochlorperazine and the indicated drugs. Cells were incubated for 72 h with DMSO or drug combinations. Afterward, cells were stained with SYTOX Red, and live cells were counted by flow cytometry. The highest single-agent (HSA) synergy model was used for evaluation. Data from three independent biological replicates are presented. Corresponding raw data are shown in Supplementary Fig. 10.

Our studies indicate that clathrin- and dynamin-2 (DNM2)-dependent endocytosis is essential for antigen-induced BCR internalization and the survival of ABC-DLBCL cells (Fig. 5C). This led us to hypothesize that inhibition of BCR endocytosis could serve as a therapeutic strategy for DLBCL treatment. The clathrin- and DNM2-dependent endocytosis pathway can be inhibited by phenothiazine derivatives, used for decades as anti-psychotic or anti-emetic agents [35]. To investigate the effect of phenothiazines on CME and BCR internalization, we first confirmed the inhibition of transferrin receptor internalization in prochloroperazine (PCH) or chlorpromazine (CPZ)-treated cells (Supplementary Fig. 7). Next, we analyzed the influence of phenothiazine derivatives on BCR endocytosis. Both PCH and CPZ increased surface BCR levels in a dose -dependent manner in ABC-type U2932 and HBL1 cells, but not in GCB-type OCI-LY19 (Fig. 7A and Supplementary Fig. 9). More importantly, cross-linking or OVA-induced BCR internalization was markedly attenuated by PCH or CPZ (Fig. 7A, B and Supplementary Fig. 9). Consistent with these results, phenothiazines also suppressed the transcription of NFκB-dependent genes in U2932 cell line in a dose-dependent manner (Fig. 7C).

Collectively, these results demonstrate that autoantigen-induced BCR internalization and subsequent NFκB activation can be pharmacologically inhibited by phenothiazine derivatives, suggesting that these compounds may suppress DLBCL growth through interference with antigen-driven BCR endocytosis.

### Phenothiazine derivatives inhibit DLBCL cell growth and synergize with BCR pathway inhibitors

We next assessed the toxicity of PCH and CPZ in a panel of ABC-DLBCL (U2932, RIVA, HBL1, and TMD8) and GCB-DLBCL (DHL4, DHL6, LY7, and LY19) cell lines. While all cell lines were sensitive to high PCH and CPZ doses (10 μM), U2932 and RIVA (ABC-DLBCL, CD79B-WT lines) exhibited markedly higher sensitivity to phenothiazine derivatives than HBL1 and TMD8 (ABC-DLBCL, CD79B-mutants) and all tested GCB-DLBCL cell lines (Fig. 7D).

To test the activity of phenothiazine derivatives in vivo, we generated U2932 xenografts in NSG mice. Treatment with PCH significantly inhibited tumor growth in this model (Fig. 7E).

Given the putative switching to tonic BCR signaling following DNM2/CME inhibition in kinome studies (Fig. 6C), we hypothesized that blocking kinases mediating tonic signaling would synergize with CME inhibition. For this reason, we evaluated the cytotoxic effects of combined inhibition of BCR internalization with SYK/PI3K blockade in DLBCL cell lines. For these studies, we chose HBL1, TMD8, and OCI-LY19 cell lines. HBL1 was shown to reprogram its BCR signaling to tonic mode after DNM2 inhibition, and both HBL1 and TMD8 were moderately sensitive to resistance to phenothiazine derivatives. Autoantigen-independent OCI-LY19 was included as a negative control. Treatment of HBL1 and TMD8 cells with PCH sensitized them to SYK inhibitors (Fostamatinib and Entospletinib) and a PI3K inhibitor (Idelalisib), whereas the effect on OCI-LY19 cells was marginal (Fig. 7F and Supplementary Fig. 10).

## Discussion

In this study, we employed precise genomic editing to modify BCR specificity and control BCR endocytosis, membrane-cytosol trafficking, and signaling in DLBCL cells. We demonstrate that, similar to normal B-lymphocytes, DLBCL cells internalize the BCR complex rapidly after antigen engagement. The complex is delivered to the endolysosomal compartment, where it forms active BCR signalosomes. When the antigen specificity is altered and BCRs are unable to bind native autoantigens, BCRs are no longer internalized. As a consequence, receptor density in ABC-DLBCL cells increases, and the formation of intracellular signaling complexes is decreased.

These observations suggested that blocking clathrin-mediated endocytosis (CME) might have similar consequences as blocking BCR–antigen engagement, while being more feasible therapeutically. CME is a tightly orchestrated process that links receptor recognition to dynamic membrane remodeling. The adapter complex AP-2 binds canonical YxxΦ motifs within cytoplasmic receptor tails, including those of the B-cell receptor (BCR), and recruits clathrin to initiate coated pit formation [36]. Together with EPS15 and its paralog EPS15L1, AP-2 forms a nucleation module with Epsin and FCHO proteins that drives membrane invagination [37, 38]. As the coat matures, clathrin heavy and light chains assemble into a lattice, the light chain coupling the process to actin dynamics [39]. BAR-domain proteins such as amphiphysin and endophilin subsequently shape the vesicle neck, recruit Dynamin-2, and promote scission of the invaginating vesicle [40]. This coordinated sequence ensures precise temporal and spatial control of receptor internalization. Of note, in prior CRISPR/Cas9 screening studies, CME-related genes (AP-2, clathrin, EPS15, endophilin, dynamin-2) were crucial for DLBCL cell survival (Supplementary Fig. 4).

Although the CME process is complex and involves orchestrated cooperation of multiple proteins, vesicle scission appears a critical step that can be therapeutically blocked. Indeed, blocking endocytosis in HBL1 cells with a dominant-negative, catalytically inactive DNM2 mutant decreased the formation of endolysosomal BCR-TLR9 signaling supercomplexes and attenuated NFκB activity. Surprisingly, DNM2 inhibition simultaneously led to activation of multiple kinases involved in tonic BCR signal generation. These observations suggest that when the autoantigen is present and available, but endocytosis is blocked, BCRs are cross-linked on the surface and the signaling is rewired to mimic a tonic signal. Surprisingly, blockade of DNM2 triggered activation of additional kinases with potentially compensatory functions. For example, CME inhibition was associated with the activation of ephrin (Eph) receptor tyrosine kinases. Eph receptors mediate bidirectional tumor-microenvironment communication, regulating tumor cell proliferation, migration, invasion, angiogenesis, and metastasis in vivo [41, 42]. These observations identify potential resistance mechanisms that may limit the efficacy of therapeutic strategies based on DNM2 inhibition. Consistent with this, the combination of PI3Kδ or SYK inhibitors (tonic signal antagonists) synergized with DNM2 inhibition. Since the role of Eph receptors in DLBCL has not been defined, these findings prompt further mechanistic studies.

Phenothiazines are anti-psychotic and anti-emetic drugs [43] acting as antagonists of dopamine receptors, but are also potent inhibitors of dynamin, making them effective at blocking CME and receptor internalization [44–48]. Consistent with this mechanism, we found that phenothiazines dose-dependently suppressed BCR internalization and downstream NFκB activation in DLBCL cells. Importantly, their cytostatic and cytotoxic effects were most pronounced in ABC-type DLBCL, which depends on chronic BCR–autoantigen engagement, whereas GCB-type lines were relatively resistant. These results strongly suggest that the therapeutic effect of phenothiazines arises primarily from inhibition of antigen-driven BCR endocytosis rather than from nonspecific cytotoxicity, providing a clear mechanistic rationale for their use in this study.

Phenothiazines administered at anti-emetic doses demonstrate an acceptable safety profile, supporting their potential repurposing as a therapeutic option for DLBCL. According to published calculations [49], a dose of 5 mg prochlorperazine/kg/day in mice corresponds to approximately 0.405 mg/kg/day for humans (24.3 mg/day for a 60 kg person). The recommended clinical dose of prochlorperazine for preventing nausea and vomiting is 30 mg daily [50]. Consistent with this, prochlorperazine in our experiments was used at the therapeutically available dose range and exhibited a significant tumor growth-inhibitory effect in mice. Therefore, achieving an anti-tumor effect in human subjects is likely feasible.

In our in vitro analyses, phenothiazines effectively dampened BCR internalization and markedly decreased proliferation of the CD79B wild-type U2932 and RIVA cells, whereas CD79B-mutant HBL1 and TMD8 cells were less sensitive. These observations suggest that the DLBCL cells with wild-type BCRs are more prone to internalization and thus, more susceptible to endocytosis inhibitors. Mechanistically, mutations in the CD79B subunit of BCR are associated with increased BCR surface expression [13]. Moreover, the membrane-proximal ITAM YxxØ motif in CD79B, a common mutation target in ABC-DLBCL, is crucial for the binding of adapter protein 2 (AP-2), the primary mediator of receptor endocytosis via clathrin-coated pits, suggesting that CD79B may regulate BCR internalization [36]. Genetic analyses indicate that 94.2% of CD79A/B mutations are heterozygous, meaning that some CD79A/B heterodimers forming the BCR will consist of the wild-type subunits. We therefore hypothesize that ABC-DLBCL cells with CD79A/B mutations possess two pools of BCRs: a wild-type BCR that can undergo self-antigen-induced internalization and trigger signaling with TLR9 from the endolysosomal compartment, and a BCR with mutated CD79A/B, less susceptible to CME – mediated internalization and remaining on the surface, initiating tonic BCR signaling (Supplementary Fig. 11). These findings suggest a potential shift to tonic BCR signaling when BCR internalization is blocked, particularly in the CD79B-mutant cells. Therefore, inhibiting both BCR internalization and tonic BCR signaling could represent an effective strategy for autoantigen-dependent cells with CD79A/B mutations. Indeed, the combination of phenothiazines with proximal inhibitors of BCR signaling demonstrated significantly greater cytotoxicity than monotherapy.

An important question arising from our study is which subgroup of DLBCL patients might benefit from a therapeutic approach that blocks CME and BCR internalization. Based on our findings, we propose that tumors with antigen-engaged, autoreactive BCRs are most likely to respond. Autoantigen-selected tumors usually utilize a restricted set of immunoglobulin heavy chain variable (IGVH) gene segments, most notably VH4-34 and VH3-7, which together account for approximately 39% of cases [19]. VH4-34-encoded BCRs confer intrinsic autoreactivity and sustain chronic-active BCR signaling, making tumor survival dependent on continuous autoantigen engagement. In this context, our findings suggest that inhibition of BCR endocytosis may be particularly effective in DLBCL cases harboring VH4-34⁺ BCRs or other autoreactive specificities.

In summary, we provide genetic, proteomic, and functional evidence that autoantigen-induced endocytosis of the BCR receptor is a key mechanism supporting BCR signaling and the survival of DLBCL cells with autoantigen-specific BCRs. Furthermore, we demonstrate that blocking endocytosis should be considered a rational therapeutic strategy in this group of lymphoid malignancies. The well-defined toxicity, pharmacokinetics, and pharmacodynamics of phenothiazines may facilitate design of clinical trials aimed at repurposing these drugs.

## Materials and methods

Complete descriptions are provided in the Supplementary Materials and Methods.

### Genomic modification

CRISPR/Cas9-mediated homologous recombination (HR) was used to generate knock-in (KI) modifications. Double-strand breaks (DSBs) at defined genomic loci were induced using the pX330-U6-Chimeric_BB-CBh-hSpCas9 plasmid (Addgene #42230), encoding Cas9 and a guide RNA (gRNA). For KI, pX330 plasmids were co-electroporated with a donor plasmid containing left and right homology arms (200–400 bp each) flanking the insert sequence. All templates included silent mutations preventing Cas9/gRNA re-targeting. Homology arms and KI sequences were synthesized as gBlocks Gene Fragments (IDT) and cloned into pSC-B-amp/kan (Agilent) for sequence verification.

To modify BCR specificity, the same approach was used to replace the hypervariable regions (HVRs) of immunoglobulin heavy and light chains with ovalbumin (OVA)-specific HVRs derived from OBI Rag1−/− mice [29]. The donor plasmids contained, from 5′ to 3′, a left homology arm, fluorescent protein (emGFP for IgH or mTurquoise2 for IgL), a self-cleaving F2A sequence, the OVA-specific HVR, and a right homology arm. Correctly modified cells were identified by fluorescence and isolated by flow cytometry.

For doxycycline-inducible expression of wild-type or K44A-mutant dynamin-2 (DNM2) and for surface expression of the mCD8a–OVA fusion, coding sequences were cloned into the transposon-based pSBtet-Bla vector (Addgene #60510). Stable integration was achieved by co-electroporation with the transposase plasmid pCMV(CAT)T7-SB100 (Addgene #34879), followed by blasticidin selection (10 μg/mL).

Electroporation was performed using the Neon Transfection System (Thermo Fisher Scientific) according to cell line–specific parameters (Supplementary Table 3).

### In vivo experiments

For in vivo assessment of prochlorperazine (PCH) activity, 5 × 10⁶ U2932 cells mixed with 30% Matrigel Matrix (Corning, Corning, NY, USA, #354230) were injected subcutaneously into 8–12 week old female NOD.Cg- *Prkdc*^*scid*^*Il2rg*^*tm1Wjl*^ (NSG) mice (Animalab, Poznan, Poland). All procedures were approved by the II Local Ethical Committee for Experiments on Animals in Warsaw, Poland (approval No WAW2/129/2023) and conducted in accordance with Directive 2010/63/EU. Mice were housed in specific pathogen-free conditions in individually ventilated cages under a 12-h light/dark cycle with ad libitum access to food and water. When tumors reached ≥100 mm³, 15 mice were randomized into three groups (*n* = 5 per group) with similar mean tumor volumes and treated with 4 mg/kg PCH, 8 mg/kg PCH, or vehicle (H₂O) intraperitoneally for 15 consecutive days. Tumor growth was measured with digital calipers. After the final dose, mice were euthanized.

### Statistical analysis

Statistical analyses were performed using GraphPad Prism 9.5.1 (GraphPad, Inc., La Jolla, CA, USA). Statistical tests are described in the figure legends. For comparisons between two groups, a two-tailed Student’s t-test was used. For in vivo experiments with mice, a one-sided Student’s t-test was applied. Testes were selected based on data distribution. *P* values < 0.05 were considered statistically significant, with significance denoted as follows: *P < 0.05; **P < 0.01; ***P < 0.001; and ****P < 0.0001. Data are presented as mean or median ± SD and are derived from number of independent biological replicates in experiment or mice, specified in figure legends. No statistical method was used to predetermine sample size for animal studies; instead, the size was chosen empirically based on our prior experience with calculating experimental variability. In the in vivo studies, animals were randomized based on size of developed tumors to generate groups with equal average tumor sizes, and investigators were not blinded to the group allocation during the experiment or when assessing the outcome.

## Supplementary information

Supplementary Information

Supplementary File 1

Supplementary File 2

Supplementary File 3

## Data Availability

The data generated in this study are available within the article and its supplementary data files. Other data that support this study and script to reproduce the analyses are available from the corresponding author upon reasonable request.
