# Supplementary material for: Inhibition of autoantigen-induced B-cell receptor (BCR) internalization as a therapeutic strategy in diffuse large B cell lymphoma (DLBCL)
Source: Cell Death Dis. 2026 Feb 11;17(1):216. doi: 10.1038/s41419-026-08446-1 (PMC12921016; doi:10.1038/s41419-026-08446-1)
Supplement: Supplementary file 1 — Supplementary Information [file 41419_2026_8446_MOESM1_ESM.pdf]

Supplementary Information

Inhibition of autoantigen-induced B-cell receptor (BCR) internalization as a therapeutic strategy in Diffuse Large B Cell Lymphoma (DLBCL)

Patryk Górniak[1], Anna Polak[1], Anna Rams[1], Kristyna Kupcova[2], Eliza Głodkowska-Mrówka[1], Zofia Pilch[3], Marta Miączyńska[4], Dominika Nowis[5], Jakub Gołąb[3], R. Eric Davis [6], Ondrej Havranek[2,7], Przemysław Juszczyński[1]

Affiliations

[1] Institute of Hematology and Transfusion Medicine, Warsaw, Poland

[2] BIOCEV, First Faculty of Medicine, Charles University, Prague, Czech Republic

[3] Department of Immunology, Medical University of Warsaw, Poland

[4] International Institute of Molecular and Cell Biology, Warsaw, Poland

[5] Laboratory of Experimental Medicine, Medical University of Warsaw, Poland

[6] Department of Lymphoma and Myeloma, The University of Texas MD Anderson Cancer Center, Houston, USA

[7] First Department of Medicine – Hematology, First Faculty of Medicine, Charles University and General University Hospital, Prague, Czech Republic

Materials and methods

Cell lines and culture conditions

For our study, we used the following DLBCL cell lines: SU-DHL4 (RRID: CVCL_0539; DSMZ, #ACC 495), SU-DHL6 (RRID: CVCL_2206; DSMZ, #ACC 572), OCI-LY7 (RRID: CVCL_1881; DSMZ, #ACC 688), OCI-Ly19 (RRID: CVCL_1878; DSMZ, #ACC 528), U-2932 (RRID: CVCL_1896; DSMZ, #ACC 633), HBL-1 (RRID: CVCL_4213; Applied Biological Materials, #T8204), and TMD8 (RRID: CVCL_A442; Cytion Biosciences, #305729). All cell lines have been routinely tested for mycoplasma contamination. Cells were cultured at 37°C with 5% CO_2 in RPMI media with L-glutamine (Sigma Aldrich, St. Louis, MO, USA) supplemented with: 10% fetal bovine serum (FBS) (Sigma Aldrich), 20 mM HEPES buffer (Lonza, Basel, Switzerland), 1 mM sodium pyruvate (Sigma Aldrich), and penicillin/streptomycin (final concentration 50 U/mL and 50 U/mL, respectively; Sigma Aldrich).

Chemicals and inhibitors

The following chemicals and inhibitors were used in the study: Prochlorperazine (Selleckchem, Houston, TX, USA, #S4631); Prochlorperazine Malate CRS - for *in vivo* studies (EDQM, Strasbourg, France, #P3200000); Chlorpromazine (Selleckchem, #S2456); Fostamatinib (Selleckchem, #S2206); Entospletinib (Selleckchem, #S7523); Idelalisib (MedChemExpress, Monmouth Junction, NJ, USA, #HY-13026).

Knock-in experiments

CRISPR/Cas9-mediated homologous recombination (HR) was used to generate knock-in (KI) modifications. DNA double-strand breaks (DSBs) at the desired genomic loci were introduced using pX330-U6-Chimeric_BB-CBh-hSpCas9 (pX330; Addgene, Watertown, MA, USA, #42230), which encodes both Cas9 and a guide RNA (gRNA) [1]. For KI, pX330 plasmids were co-electroporated with

an HR template plasmid containing a left homology arm (LHA, 200–400 bp), the insert sequence, and a right homology arm (RHA, 200–400 bp). All homology arms and inserts were designed with silent mutations to prevent re-targeting by Cas9/gRNA. Homology arms and KI sequences were synthesized as gBlocks Gene Fragments, cloned into pSC-B-amp/kan (StrataClone Ultra Blunt PCR Cloning Kit, Agilent, Santa Clara, CA, USA), and sequence-verified. The HR template plasmids were constructed by combining the LHA and RHA as a single gBlock, separated by a cassette with two type IIS restriction enzyme sites for seamless KI sequence insertion. Specific details and sequences for all HR templates can be found in the Sequences section in the Supplement.

To modify BCR specificity, the same KI approach was used to replace the BCR hypervariable region (HVR). Two pX330 plasmids induced DSBs flanking the original HVR, and a repair template plasmid provided the new ovalbumin (OVA)-specific HVR and a fluorescent protein (FP) marker. OVA-specific HVRs were based on published sequences from OBI Rag1−/− mice with OVA-reactive B cells [2] . To recreate the complete OVA HVR, we incorporated the 5′ portions of the full HVRs (including the leader sequence and V intron) based on the mouse reference sequence predicted by IMGT/V-QUEST.

The repair template plasmid, from 5′ to 3′, included: LHA, FP, a 58-amino acid F2A sequence, HVR, and RHA. The FP cDNA was positioned with a Kozak sequence for in-frame translation (emGFP for H-HVR, mTurquoise2 for L-HVR). The F2A sequence ensured optimal separation of FP and HVR. The inserted HVR included its leader sequence and V intron. Following HR-mediated KI, the modified genomic sequence spanned from the V region translation initiation site to the 3′ end of the J segment, with the endogenous IgH or IgL promoter driving the expression of the FP (as a modification marker) alongside the separate IgH or IgL containing the replaced HVRs. In all experiments, we used control HVR replacements in which the original (endogenous) HVR was inserted along with the fluorescent marker. BCR HVR sequences for each cell line were previously published (Havranek et al., 2017) [3] . The CRISPR/Cas9 target sequences used for HVR replacement, and the basic characteristics of the DLBCL cell lines used, are provided in Supplementary Table 1 and Supplementary Table 2, respectively. HA

fragments with FPs and F2A, and individual H-HVR and L-HVR fragments, are also listed in the Sequences section in the Supplement.

For dual HVR replacement, both IgH and IgL HVRs were targeted simultaneously by electroporating six plasmids (upstream and downstream CRISPR/Cas9 and HR template for each Ig chain), 4 µg each, into 1.2 million cells in 120 µL R buffer. Double HVR-replaced (GFP- and mTurquoise2-positive) cells were monitored by flow cytometry.

Inducible expression of DNM2 and mCD8-OVA

To enable doxycycline-inducible expression of wild-type or K44A mutant DNM2, synthesized coding sequences for both variants (ATG Biosynthetics) were PCR-amplified and cloned into the transposon-based pSBtet-Bla vector (Addgene #60510) using the NEBuilder® HiFi DNA Assembly Kit. For stable genomic integration, 6 µg of the resulting transposon plasmid and 4 µg of the transposase plasmid pCMV(CAT)T7-SB100 (Addgene #34879) were electroporated into 1.2 million cells using the Neon Transfection System (Thermo Fisher Scientific, Waltham, MA, USA). Transfected cells were selected with blasticidin (10 µg/mL) (Thermo Fisher Scientific).

For surface expression of the OVA peptide, a fusion construct comprising the transmembrane region of mouse CD8a and the OVA peptide (mCD8a-OVA) was cloned into the pSBtet-Bla vector. Cells were co-electroporated with this construct and the transposase plasmid using the Neon device. All nucleotide sequences are provided in the Supplement.

Transfection

Cells were transfected with plasmid DNA using the Neon electroporation system (Thermo Fisher Scientific) in 100 µL volumes. Log-phase cells were cultured with daily medium changes for three days prior to electroporation and washed once with PBS. For each electroporation, 1.2 million cells were resuspended in 120 µL of buffer R, mixed with maxiprep-purified plasmid DNA (PureLink HiPure Kit,

Thermo Fisher Scientific), and electroporated under cell line–specific conditions (Supplementary Table 3). Afterward, cells were transferred to 3 mL of pre-warmed, antibiotic-free medium.

Cell viability and growth assay

Growth rates and viability after inhibitor treatment were assessed by bead-based flow cytometry. Cells were stained with SYTOX® Red (Thermo Fisher, 1:1000). Before analysis, 10 µL of 1:10 diluted 6.0–8.0 µm polystyrene beads (Spherotech, Lake Forest, IL, USA) were added. Beads and cells were distinguished by scatter on a CytoFLEX cytometer (Beckman Coulter, Indianapolis, IN, USA). Absolute cell numbers were determined by comparing bead and cell counts. If the culture was maintained over time through passaging at known dilutions, a growth curve reflecting the increase in cell number over time was constructed. IC_{50} values were calculated using Quest Graph™ IC50 Calculator (AAT Bioquest, Pleasanton, CA, USA), and drug synergy was analyzed with SynergyFinder.

Proximity Ligation Assay (PLA)

Cells were centrifuged, resuspended in PBS, and plated on precision coverslips for 20 min at 37°C. After adhesion, cells were fixed with 4% paraformaldehyde (Sigma Aldrich) for 20 min, washed, and membranes labeled with 5 µg/ml WGA-Alexa Fluor 488 (Thermo Fisher Scientific) for 10 min. Cells were permeabilized with 0.5% Triton X-100 (Sigma) in PBS for 10 min, washed, and blocked in Duolink Blocking Buffer (Sigma Aldrich) for 30 min. Primary antibodies (see Supplementary Table 5) were diluted in Duolink Antibody Diluent and incubated overnight at 4°C. After washing, Duolink Probes (Sigma Aldrich) were added and incubated for 1 h at 37°C, followed by washes. Ligation and amplification were performed with Duolink In Situ Detection Reagents Red kit (Sigma Aldrich) per manufacturer's instructions. Cells were mounted in Prolong Gold with DAPI (Invitrogen, Carlsbad, CA, USA). Images were acquired on a Zeiss Axio Imager.Z2 fluorescence microscope and analyzed in ImageJ/FIJI.

The antibodies we used for PLA analysis were previously validated by Phelan et al. [4] and are listed in the Supplementary Table 5.

Flow cytometry-based BCR internalization assay

DLBCL cell lines (HBL1, U2932, OCI-Ly19) were stimulated with 5 µg/mL anti-human IgM F(ab')$_2$ fragments (Jackson ImmunoResearch, West Grove, PA, USA). To induce OVA-specific BCR internalization, cells were treated with either 1 µM full-length ovalbumin (Sigma Aldrich) or a biotinylated OVA 17-mer peptide (FDKLPGFGDSIEAQGGK; GenScript, Nanjing, China) pre-complexed with avidin at a 4:1 molar ratio. For stimulation, 20 µL of a 10 µM peptide-avidin complex (calculated based on avidin concentration) was added to 1 mL of cell suspension. Following stimulation, cells were placed on ice to halt internalization, stained with APC-conjugated anti-human kappa light chain antibody (Invitrogen), and analyzed by flow cytometry (CytoFLEX, Beckman Coulter). Surface BCR levels were quantified using FlowJo software.

Transferrin Internalization Assay

Cells were incubated with Alexa Fluor 488–labeled transferrin (20 µg/mL; Invitrogen) for 1 hour at 37°C to allow internalization. Following incubation, cells were placed on ice to halt further uptake, washed, and incubated with or without anti–Alexa 488 quenching antibody (Invitrogen) to distinguish internalized from surface-bound transferrin. Samples were analyzed by flow cytometry (CytoFLEX, Beckman Coulter), and data were processed using FlowJo software (FlowJo, LLC).

Flow cytometry

For surface antigen detection (mCD8-OVA and BCR), 0.5 × 10^6 cells were washed, stained with the appropriate antibody in FACS buffer (PBS with 1% FBS) for 30 min on ice, washed, and resuspended in FACS buffer. Data were acquired on a CytoFLEX cytometer and analyzed with FlowJo.

Western-Blot

Cells were washed in PBS and lysed in RIPA buffer with protease and phosphatase inhibitors (Roche) as described [5] . Proteins were separated via SDS–PAGE on 4–15% gradient gels (Bio-Rad), transferred to PVDF membranes (Millipore, Burlington, MA, USA), and blocked with 5% BSA/TBST. Membranes were incubated with primary antibodies (1:1000; Supplementary Table 1) overnight at 4°C, then with HRP-conjugated secondary antibodies. Signals were visualized using ECL (Perkin Elmer, Waltham, MA, USA) and captured with the G:Box system (Syngene, Bengaluru, India). Densitometric quantifications were performed using ImageJ software (https://imagej.nih.gov/ij/).

Real-time PCR

RNA was extracted using the GeneMATRIX Universal RNA Purification Kit (EURx, Gdansk, Poland), and cDNA was synthesized with the Transcriptor Universal cDNA Master (Roche, Basel, Switzerland). qPCR was performed using SYBR Green Master Mix on a CFX96 Real-Time System (Bio-Rad, Hercules, CA, USA). Expression levels were normalized to GAPDH using the ΔΔCT method. Primer sequences are listed in Supplementary Table 4.

Tyrosine and Serine/threonine kinase profiling

For tyrosine and serine/threonine kinase profiling, the PamStation12 with PTK and STK PamChip peptide arrays (PamGene, Wolvenhoek, Netherland) was utilized. Analysis was performed according to the manufacturer's instructions. In brief, chips were blocked with 2% BSA (Sigma-Aldrich). Proteins were extracted from fresh-frozen cell pellets using T-PER Buffer (Thermo Fisher Scientific), supplemented with 1:100 Phosphatase Inhibitor Cocktail and 1:100 Halt Protease Inhibitor Cocktail (EDTA-free, Thermo Fisher Scientific). 1 µg of protein per sample was applied to the chips with kinase buffer, ATP, and FITC-labeled antibodies.

Signal intensities were quantified using BioNavigator 6.1.42 (PamGene), expressed per 100 ms exposure, and log-transformed. A mean value of <20% for peptides with a signal >2,000 was

established to ensure quality standards. Normalization was applied, and two replicated quantifications were combined using a false discovery rate (FDR) < 1%. A *P* value < 0.05 and a >10% fold change were considered significant.

Calcium flux

For calcium flux analysis, cells were loaded with Calbryte 630AM by resuspending in RPMI with 2% FBS and 25 mM HEPES containing 10 µM Calbryte 630AM (AAT Bioquest) and 0.05% Pluronic® F-127 (Thermo Fisher Scientific). Cells were incubated for 45 min at 37°C in the dark, washed twice with DMEM containing 2% FBS, and resuspended in loading medium. For stimulation, cells were treated with 6 µg/mL $F(ab')_2$ anti-human IgM goat antibody (Jackson ImmunoResearch) or 1 µM ovalbumin (Sigma Aldrich). Calcium responses were measured in the red channel using a CytoFLEX Flow Cytometer (Beckman Coulter).

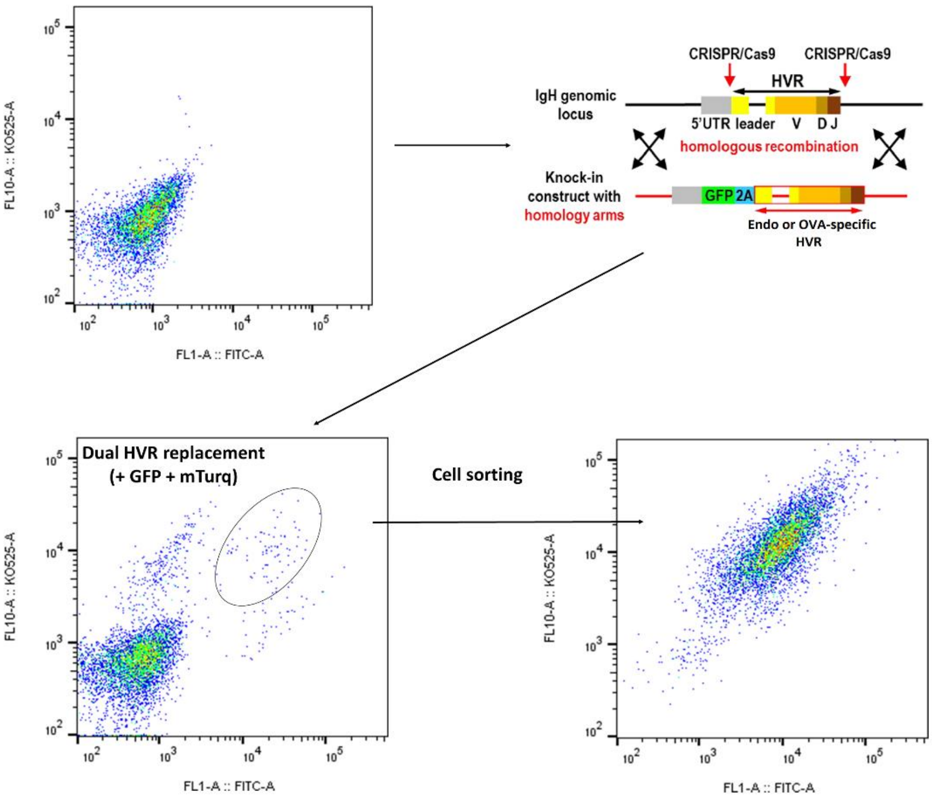

Supplementary Figure 1. Scheme illustrating the replacement of IgH hypervariable region (HVR) fragments in DLBCL cells and the associated flow cytometry gating strategy. Complementary DNA encoding a fluorescent protein (GFP for IgH), followed by sequences for a 2A peptide (which creates a break during translation), a signal peptide, and either an OVA-specific HVR or an endogenous HVR, was knocked in at the start of the immunoglobulin heavy (IgH) translation site using CRISPR-Cas9 methodology. The design for replacing the IgL HVR is similar, utilizing a CFP variant (mTurquoise2) with no D segment. Five days post-electroporation, GFP and mTurquoise2 double-positive cells were sorted for further experiments.

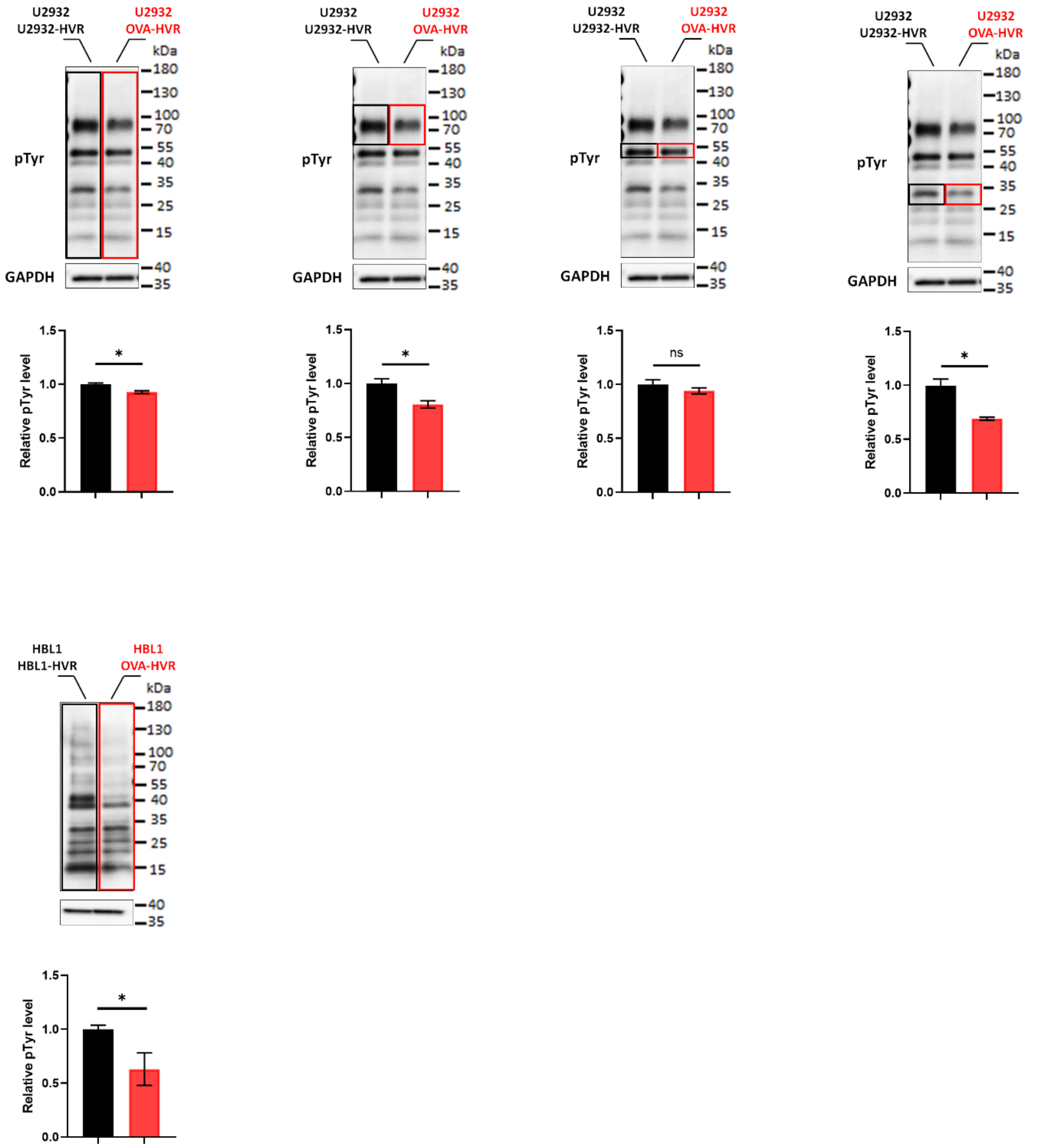

Supplementary Figure 2. Western blot analysis of tyrosine-phosphorylated proteins in cells with OVA-recognizing and endogenous HVRs. GAPDH was used as a loading control. Representative blots from two independent experiments per cell line are shown. Uncropped immunoblot images are provided in the Supplementary Material. Densitometric quantification of bands from both replicates is presented as a bar chart. Red (OVA-HVR) and black boxes (endogenous-HVR) indicate regions that were included in the densitometric analysis. P values were calculated using a two-sided t-test.

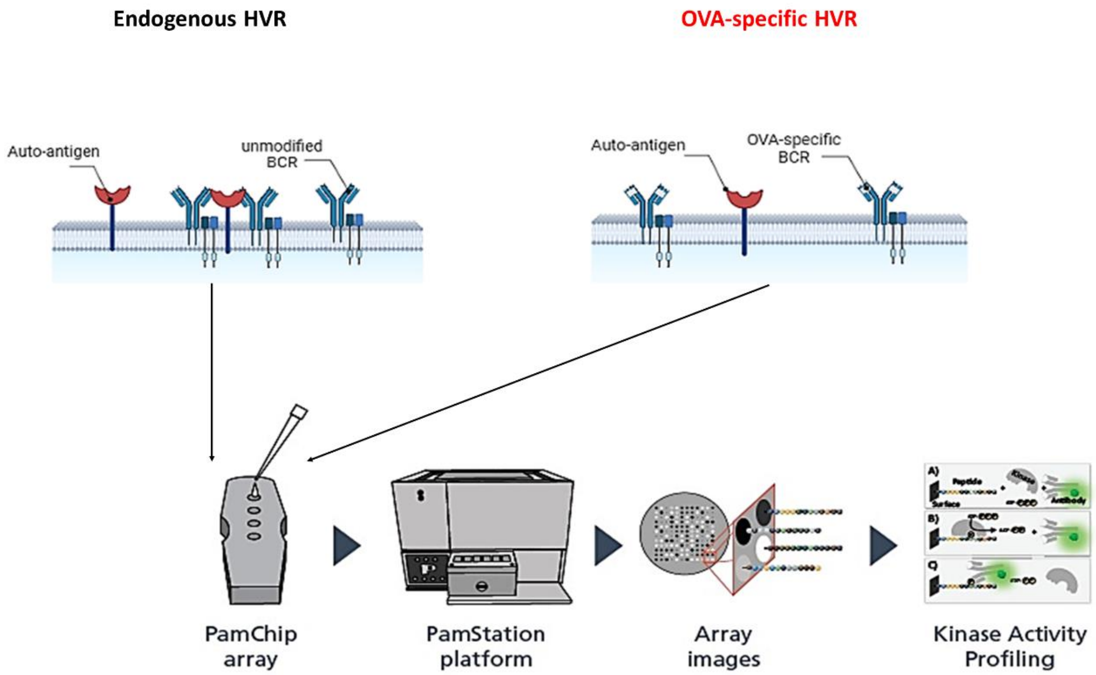

Supplementary Figure 3. Schematic presentation of experimental setup for the identification of autoantigen-dependent signaling using dual HVR-replaced DLBCL models and the PamGene platform. This fluorescent platform measures the ability of active kinases in a specimen to phosphorylate specific peptides imprinted on multiplex chip arrays. Each chip contains four arrays, with the STK and PTK arrays displaying 144 serine/threonine and 196 tyrosine immobilized peptides, respectively. Each peptide represents a 15-amino-acid sequence from putative phosphorylation sites in human proteins, derived from the literature and correlated with one or more upstream kinases. In the presence of ATP, the kinases in the sample actively phosphorylate substrates on the PamChip. Phosphorylation is detected using an antibody, while a second FITC-conjugated antibody quantifies the signal.

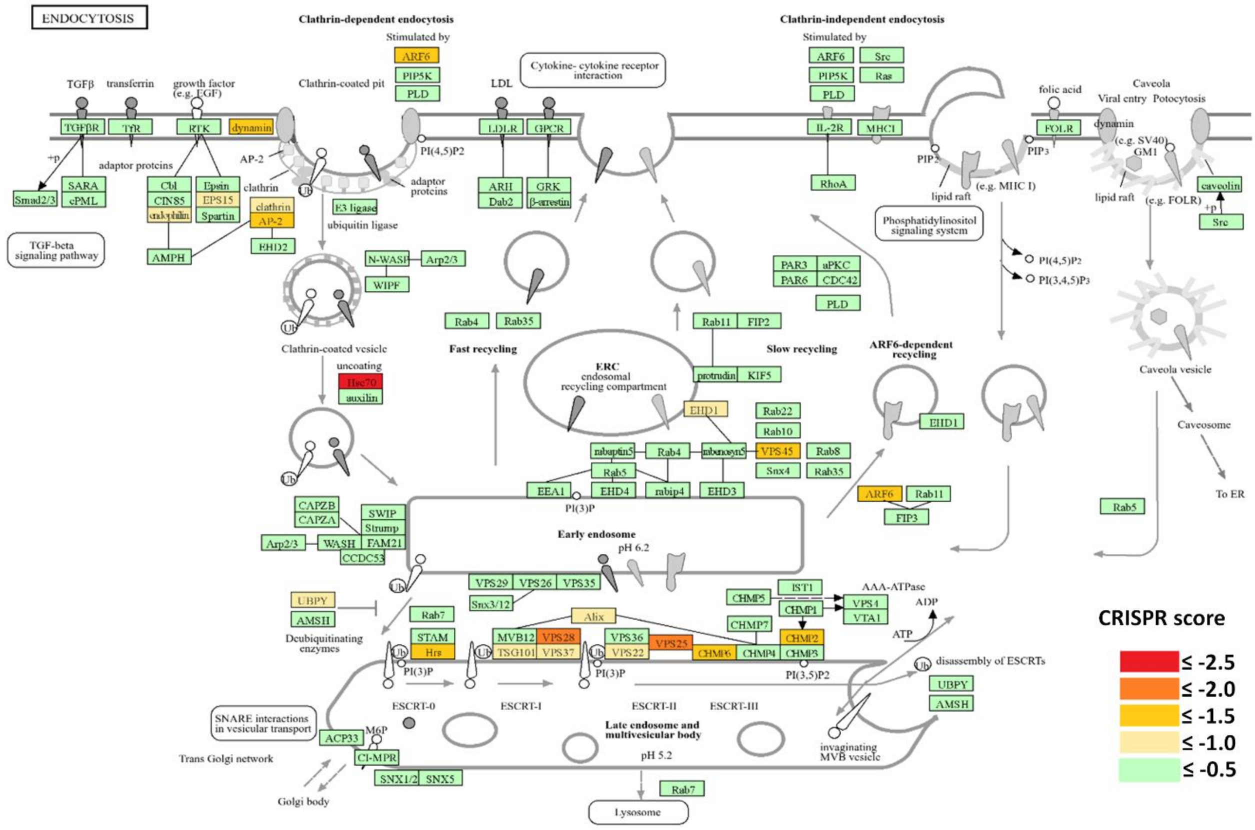

Supplementary Figure 4. Analysis of CRISPR-Cas9 screening data from Reddy et al. (2017) using the KEGG Endocytosis Human Gene Set (hsa04144). A CRISPR score ≤ 0 indicates a growth-inhibitory effect of the depletion of a particular gene.

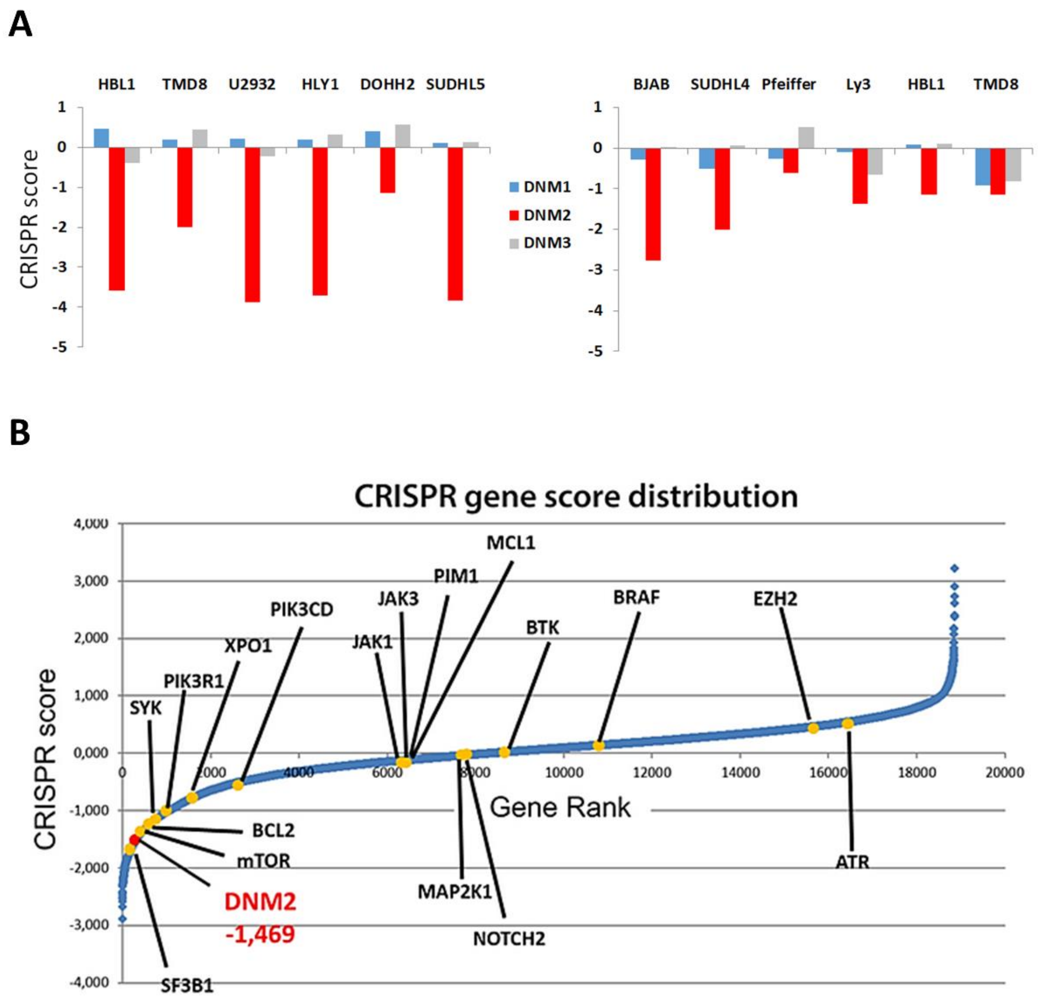

Supplementary Figure 5. CRISPR screen in DLBCL cell lines. (A) CRISPR scores for DNM1/2/3 in DLBCL cell lines, utilizing datasets from Phelan et al. (2018) for the left panel and Reddy et al. (2017) for the right panel. (B) Ranked list of CRISPR scores for the 19,032 genes targeted in the screen. Genes targeted by currently used inhibitors are highlighted in yellow, with DNM2 shown in red (data from Reddy et al., 2017). A CRISPR score < 0 indicates that CRISPR-mediated gene depletion is lethal.

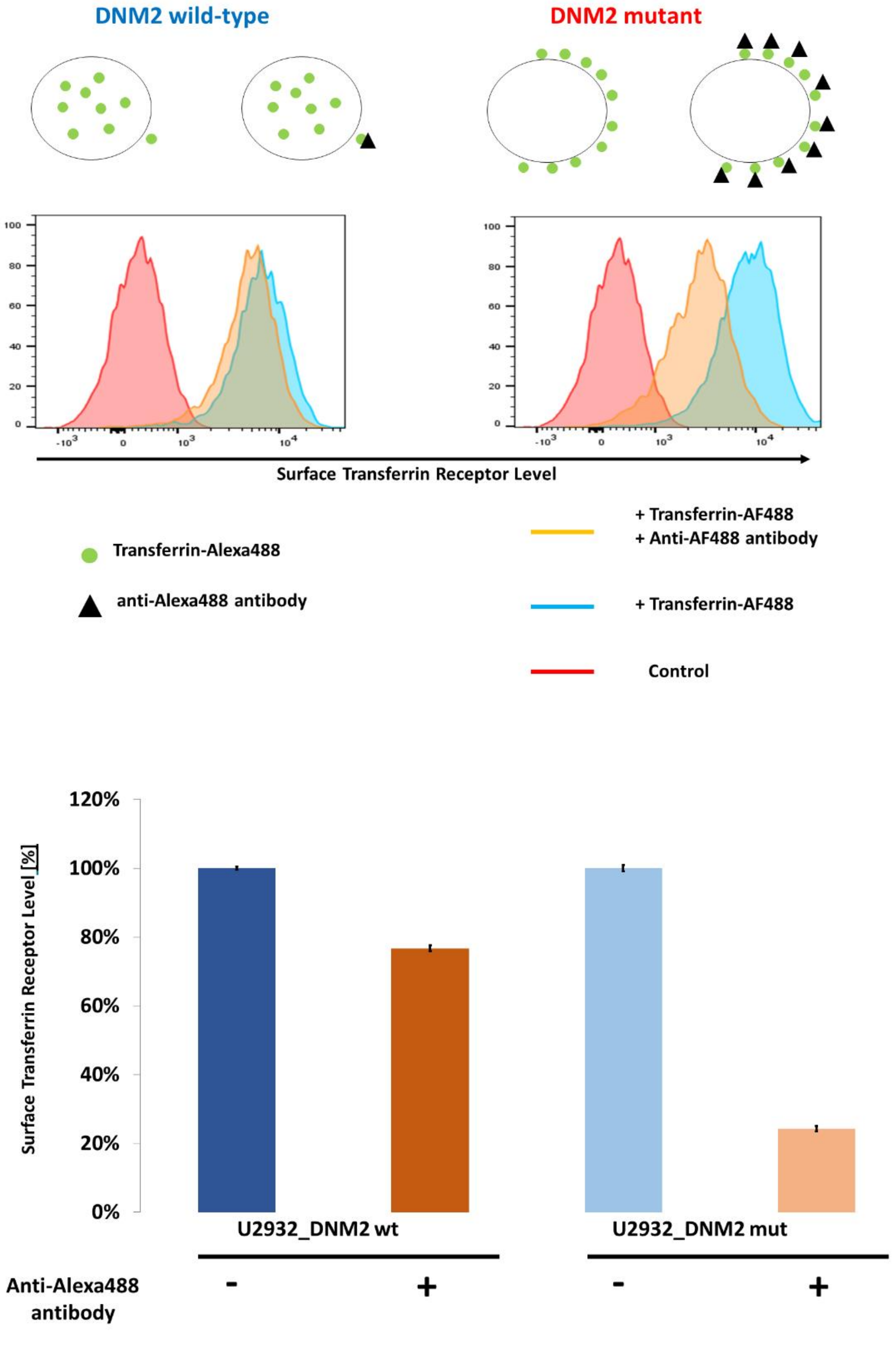

Supplementary Figure 6. Expression of DNM2mut inhibits transferrin receptor internalization in DLBCL cells. To induce DNM2 wild-type (DNM2wt) or mutant (DNM2mut) versions, U2932 cells were incubated with doxycycline (DOX) (100 ng/mL) for 24 hours. Flow cytometry-based transferrin assays were then performed, and results are presented as histograms and MFI plots (means ± SD of 2 independent replicates). Transferrin-AF488 internalization in cells expressing DNM2wt is not impaired, therefore most of the transferrin-AF488 is located inside the cell. Addition of anti-AF488 quenching antibody, does not significantly affect the signal. In cells expressing DNM2mut, transferrin-AF488 internalization is inhibited. Therefore, most of the transferrin-AF488 is located on the cell surface, and addition of anti-AF488 antibody significantly inhibits the AF488 signal.

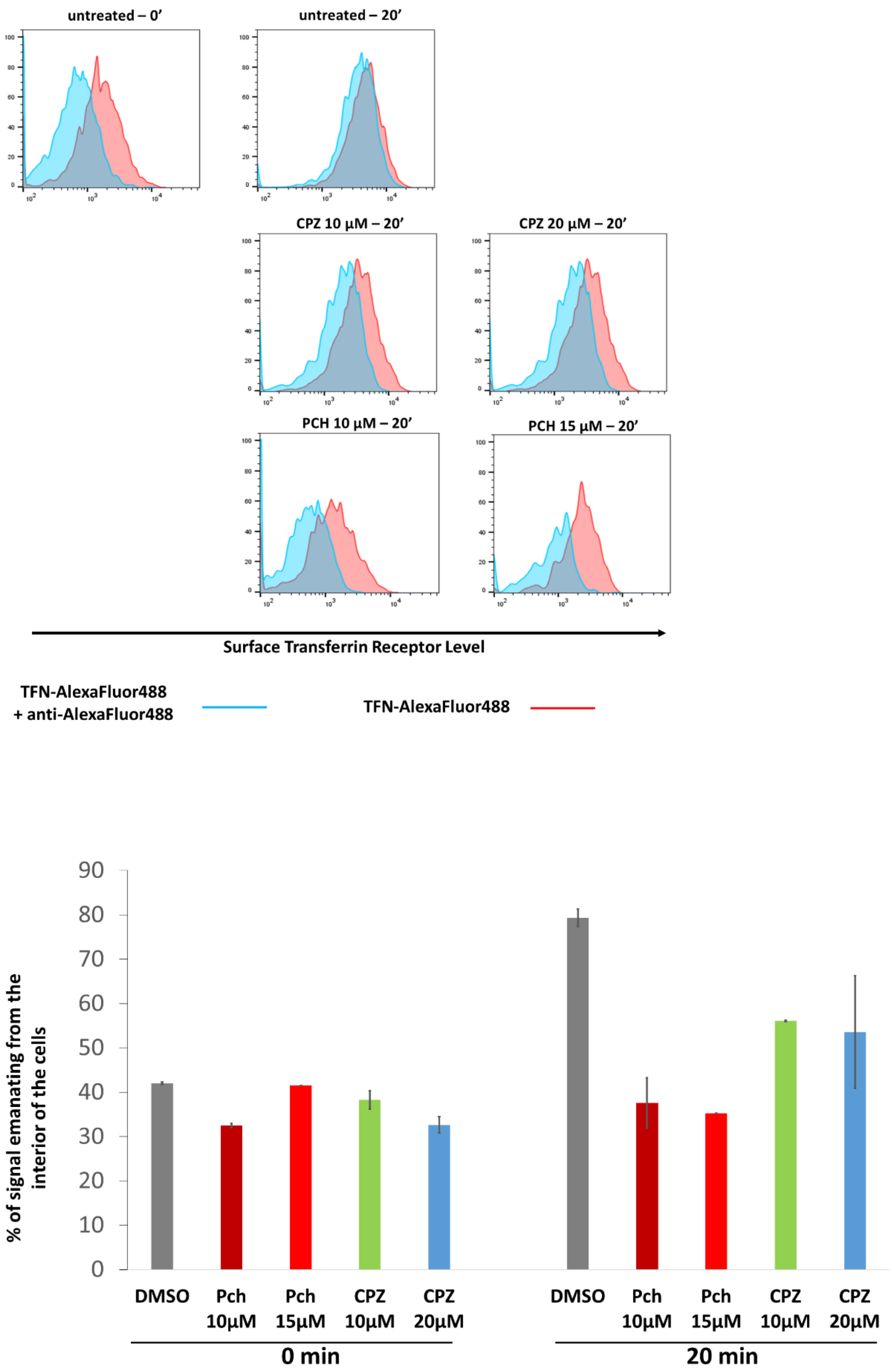

Supplementary Figure 7. Phenothiazine derivatives inhibit transferrin receptor internalization in DLBCL cells. U2932 cells were incubated with DMSO, prochlorperazine (PCH) (10 and 15 µM), or chlorpromazine (CPZ) (10 and 20 µM) for 16 hours, followed by flow cytometry-based transferrin assays. Results are presented as histograms and MFI plots (mean ± SD of 2 independent replicates).

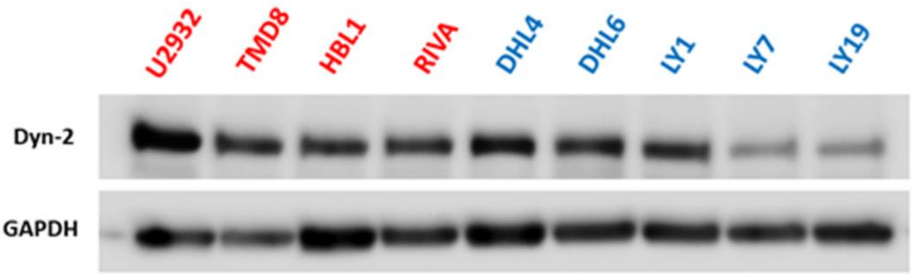

Supplementary Figure 8. Western blot analysis demonstrating the expression of Dynamin-2 in DLBCL cell lines. GAPDH was used as a loading control.

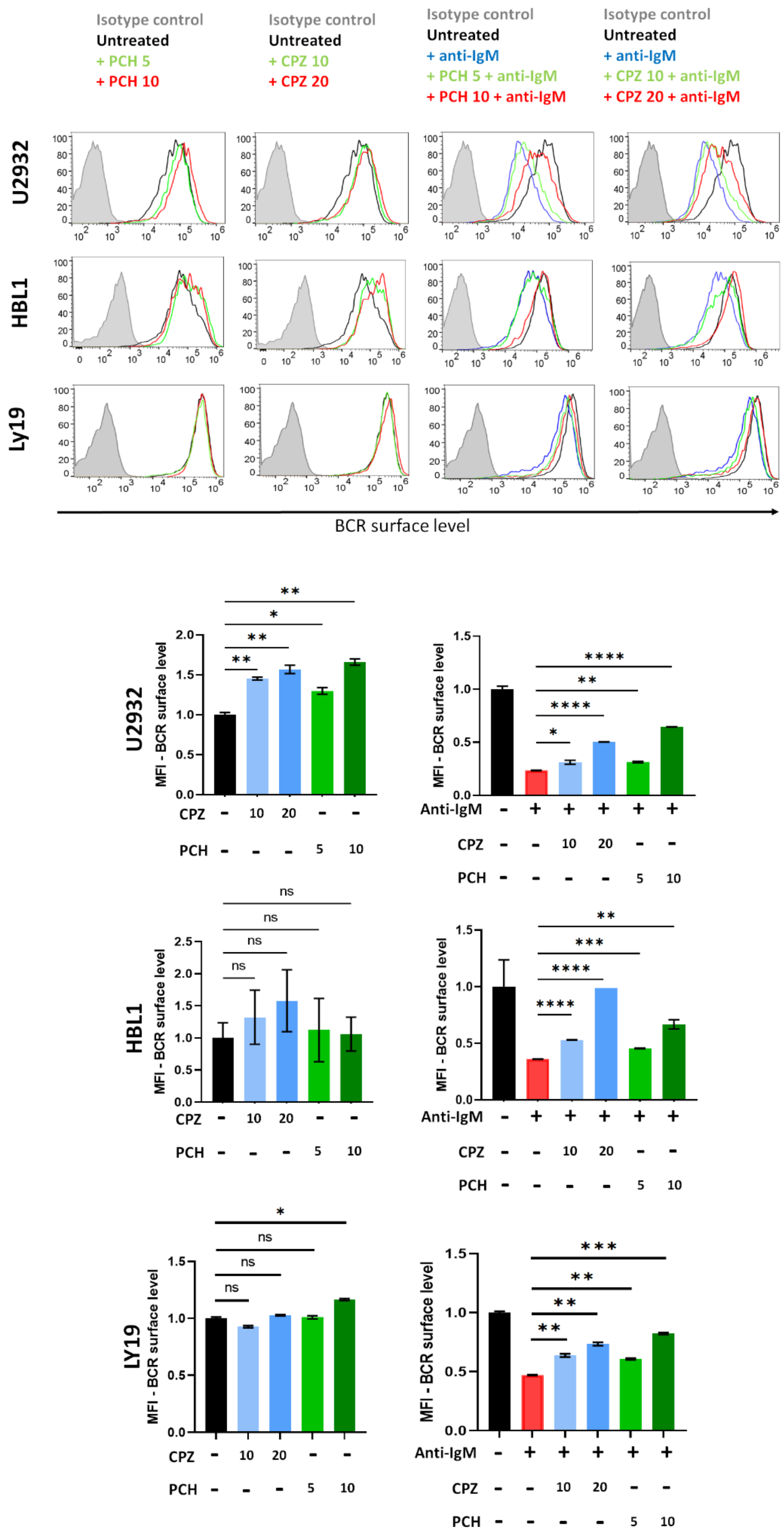

Isotype control
Untreated
+ PCH 5
+ PCH 10
Isotype control
Untreated
+ CPZ 10
+ CPZ 20
Isotype control
Untreated
+ anti-IgM
+ PCH 5 + anti-IgM
+ PCH 10 + anti-IgM
Isotype control
Untreated
+ anti-IgM
+ CPZ 10 + anti-IgM
+ CPZ 20 + anti-IgM
U2932
HBL1
Ly19
BCR surface level
MFI - BCR surface level
CPZ
PCH
Anti-IgM
ns
LY19

Supplementary Figure 9. BCR internalization in phenothiazine derivative-treated DLBCL cells. U2932, HBL1 and LY19 cells were treated with DMSO, prochlorperazine (PCH) (5 and 10 μM), or chlorpromazine (CPZ) (10 and 20 μM) for 16 hours and then stimulated with anti-IgM (6 μg/mL for 1 hour) to induce BCR internalization. Surface BCR levels were assessed by flow cytometry, and the results are presented as histograms and mean fluorescence intensity (MFI) plots (means ± SD of two independent replicates). *P* values were calculated by using two-sided unpaired *t* test

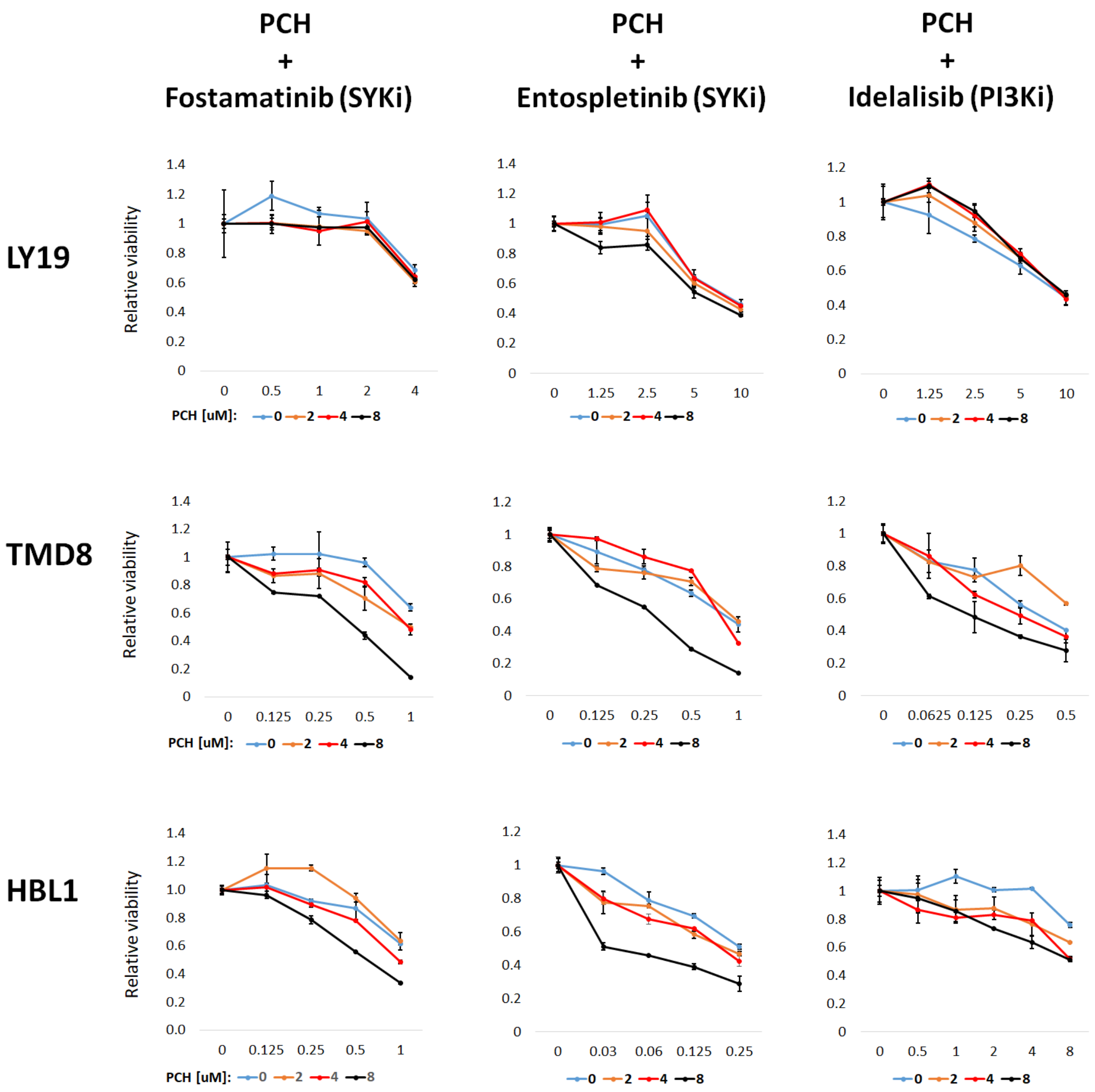

Supplementary Figure 10. Drug synergy analysis in LY19, TMD8, and HBL1 cells treated with prochlorperazine in combination with the indicated drugs. Cells were incubated for 72 hours with DMSO or the respective drug combinations. After incubation, cells were stained with SYTOX Red, and live cells were quantified by flow cytometry. Raw dose–response curves are shown. Data from three independent biological experiments are presented, with error bars representing standard deviation (SD).

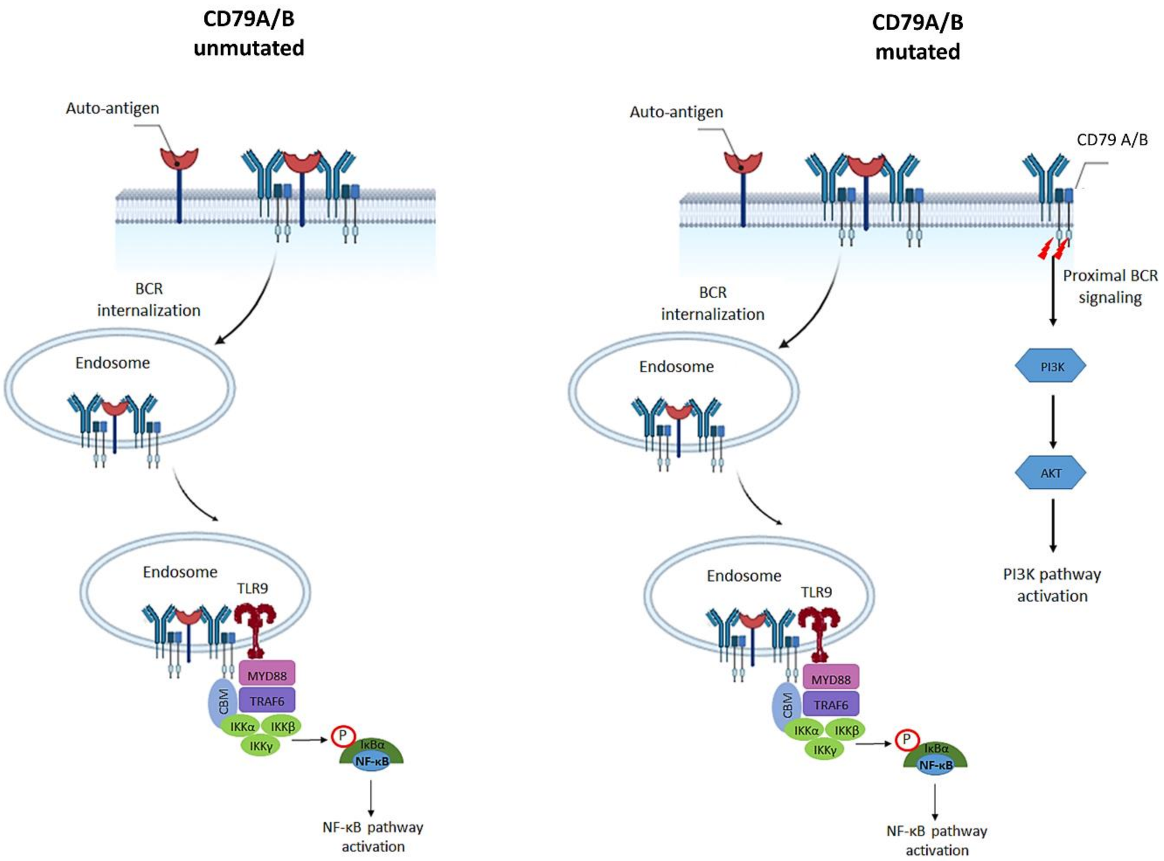

Supplementary Figure 11. Hypothetical modes of BCR signaling in DLBCL. In the ABC-DLBCL subtype, mutations in CD79A/B disrupt BCR internalization, resulting in two distinct pools of BCRs in cells harboring heterozygous mutations: a completely wild-type BCR capable of autoantigen-induced internalization, leading to the assembly of the oncogenic BCR-TLR9 complex, and a BCR with mutated CD79A/B that remains on the cell surface, sustaining tonic BCR signaling. In ABC-DLBCL cells with wild-type CD79A/B, all BCRs can respond to autoantigens; thus, BCR signaling is predominantly reliant on internalization and the assembly of the BCR-TLR9 complex. In contrast, autoantigen-independent (GCB-type) cells primarily utilize tonic BCR signaling, with PI3K (phosphatidylinositol 3-kinase) serving as the major effector.

Supplementary Table 1. Sequences of genomic target sites used for knock-in (KI) experiments.

Name	Target Sequence (5' to 3')	Location
HBL1_HV_07	GAAGTGCTTTCTGAGAGTCATGG	V 5' UTR
HBL1_HV_04	AAACCAGGAGAGACGTTGTGAGG	beginning of post J intron
HBL1_LV_01	TGGAGAAGAGCTGCTCAGTTAGG	V 5' UTR
HBL1_LV_04	TCGTGAGATTTTAGTGCCATTGG	beginning of post J intron
Ly19_HV_08	CAGAGGACTCACCATGAAGTTGG	beginning of V
Ly19_HV_06	AAAGTAAATGAGACGTTGTGAGG	beginning of post J intron
Ly19_LV_01	AGAGATTTTCCCTGAAGTTCCGG	V intron
Ly19_LV_03	ATATATCACTTCATAGACACAGG	beginning of post J intron
U2932_HV_02	GCAAGAAAATGAAGCACCTGTGG	beginning of V
U2932_HV_03	AAAGCAGGAGAGAGGTCGTGAGG	beginning of post J intron
U2932_LV_01	TGGAGAAGAGCTGCTCAGTTAGG	V 5' UTR
U2932_LV_04	TAGATCACTTCATAGACACAGGG	beginning of post J intron

Supplementary Table 2. Basic characteristics of DLBCL cell lines used.

Cell line	Ig Heavy isotype	Ig light isotype
HBL1	M	κ
U2932	M	κ
OCI-Ly19	M	κ

Supplementary Table 3. Neon electroporation conditions.

Cell line	Electroporationconditions
HBL1	1200 V, 20 ms, 2 Pulses, R buffer
U2932	1400 V, 20 ms, 1 Pulse, R buffer
OCI-Ly19	1500 V, 10 ms, 3 Pulses, R buffer

Supplementary Table 4. Primers used in gene expression analysis.

**TNAIFP3_ For**	5'-ATGATACTCGGAACTGGAATG-3'
**TNAIFP3_ Rev**	5' - ATGACAATGATTGGCCTTCTG-3'
**BCL2L1_For**	5' - GGATTTGAATCTCTTTCTCTCC-3'
**BCL2L1_Rev**	5' - CAACCACCAGCTCCCGGT- 3'
**CD40_For**	5'- AACAGGCAGGCACAAACAAGACTG-3'
**CD40_Rev**	5'- TGGCAAACAGGATCCCGAAGATGA-3'
**BCL2A1_For**	5' - CAGAAGATGACAGACTGTGAA-3'
**BCL2A1_Rev**	5' - TCCAAGCATGACTTCAGATTC-3'
**NFkBiz_For**	5' - ATGGTGACACGTTCCTTCATA- 3'
**NFkBiz_Rev**	5' - CTGCACAATGAGATGCTGATT- 3'
**TNFα_For**	5' - AGGACGAACATCCAACCTTCCCA – 3'
**TNFα_Rev**	5' - TTTGAGCCAGAAGAGGTTGAGGG – 3'
**IL6_For**	5' - TTCTCCACAAGCGCCTTCG – 3'
**IL6_Rev**	5' - CTGAGATGCCGTCGAGGAT – 3'
**NFKBIA_For**	5' - CCAACTACAATGGCCACACGT - 3'
**NFKBIA_Rev**	5' - TCCGGCCATTACAGGGCT – 3'
**GAPDH_For**	5' - AGCCTCCCGCTTCGCTCTCT-3'
**GAPDH_Rev**	5' - CGACCAAATCCGTTGACTCCGAC-3'

Supplementary Table 5. Antibodies used.

Antigen	Source	Catalog no.	Application
Anti-Human Kappa-APC	Thermo Fisher Scientific	MH10515	Flow cytometry
Anti-Mouse CD8a-PE	BD Pharmingen	553032	Flow cytometry
Mouse IgG3-APC	R&D systems	IC007A	Flow cytometry
Rat IgG2A- PE	R&D systems	IC006P	Flow cytometry
4G10 Platinum Anti-Phospho tyrosine	Millipore	05-1050	Western Blot
Anti phospho-IκBα (S32)	Cell Signaling	2859S	Western Blot/PLA
Anti- IκBα	Cell Signaling	9242	Western Blot
Anti phospho-CD79A (Y182)	Cell Signaling	5173	Western Blot
Anti phospho-SRC family (Y416)	Cell Signaling	2101	Western Blot
Anti phospho-AKT (S473)	Cell Signaling	9271	Western Blot
Anti-Dynamin-2	Abcam	AB65556	Western Blot
Anti-GAPDH	Millipore	MAB 374	Western Blot
Goat Anti-Human IgM, $Fc_{5\mu}$ fragment specific	Jackson Immunoresearch	109-005-129	PLA
anti-LAMP1	Santa Cruz Biotechnology	sc-20011	PLA
Anti-TLR9	Cell Signaling	2254S	PLA

Supplementary File 1. Comparison of tyrosine and serine/threonine kinase activities in HBL1 OVA-specific vs. HBL1 control cells using the chip-based phosphoproteomic PamGene platform.

Supplementary File 2. Comparison of tyrosine and serine/threonine kinase activities in K44A-DNM2 mutant vs. wild-type DNM2-expressing HBL1 cells using the chip-based phosphoproteomic PamGene platform.

SEQUENCES

Homology arms for H-HVR replacement with inserted GFP and F2A sequences

Sequences of homology arms with inserted GFP and F2A sequences were previously published by Havranek et al. Blood 2017.

Fragments of DNA were inserted into the pSC-B-amp/kan plasmid (Agilent Technologies) and used for ligation mediated assembly of repair template plasmids for H-HVR and L-HVR replacement.

The sequences are marked as follows:

Introns: GTTATAT
Exons in bold: **CCTTAAA**
GFP: ATGGTGA
F2A: GTGACAG
Splice donor and acceptor sites: GT AG
Silent changes to prevent re-targeting of repair template plasmid and modified genomic locus: ACTGG
BsmBI restriction cassette: GGAGACGCCCTCGTCTCC
BsmBI restriction sites: GAGACG and CGTCTC
Cas9/sgRNA sites: underlined

HBL-1_H_HA_GFP_F2A

AACTGTATTCCAAAATCTGTCTTTGATCCATGATCACACTTGTCTCCCAGACCAGCTCCTTCAGCACA
TTTCCTACCTGGAAGAAGAGGACTCTGGGTTTGGTGAGGGGAGGCCACAGGAAGAGAACTGAGTTCTC
AGAGGGCACAGCCAGCATACACCTCCCAGGGTGAGCCCAAAAGACTGGGGCCTCCCTCATCCCTTTTT
ACCTATCCATACAAAGGCACCACCCACATGCAAATCCTCACTTAGGCACCCACAGGAAATGACTACAC
ATTTCCTTAAATTCAGGGTCCAGCTCACATGGGAAGTGCTTTCTGAGAGTCCTGAAACTTCTGCACAA
GAACATGGTGAGCAAGGGCGAGGAGCTGTTCACCGGGGTGGTGCCCATCCTGGTCGAGCTGGACGGCG
ACGTAAACGGCCACAAGTTCAGCGTGTCCGGCGAGGGCGAGGGCGATGCCACCTACGGCAAGCTGACC
CTGAAGTTCATCTGCACCACCGGCAAGCTGCCCGTGCCCTGGCCCACCCTCGTGACCACCTTCACCTA
CGGCGTGCAGTGCTTCGCCCGCTACCCCGACCACATGAAGCAGCACGACTTCTTCAAGTCCGCCATGC
CCGAAGGCTACGTCCAGGAGCGCACCATCTTCTTCAAGGACGACGGCAACTACAAGACCCGCGCCGAG
GTGAAGTTCGAGGGCGACACCCTGGTGAACCGCATCGAGCTGAAGGGCATCGACTTCAAGGAGGACGG
CAACATCCTGGGGCACAAGCTGGAGTACAACTACAACAGCCACAAGGTCTATATCACCGCCGACAAGC
AGAAGAACGGCATCAAGGTGAACTTCAAGACCCGCCACAACATCGAGGACGGCAGCGTGCAGCTCGCC
GACCACTACCAGCAGAACACCCCCATCGGCGACGGCCCCGTGCTGCTGCCCGACAACCACTACCTGAG
CACCCAGTCCGCCCTGAGCAAAGACCCCAACGAGAAGCGCGATCACATGGTCCTGCTGGAGTTCGTGA
CCGCCGCCGGGATCACTCTCGGCATGGACGAGCTGTACAAGGTGACAGAGTTGCTGTACAGGATGAAG
CGGGCCGAGACCTACTGTCCAAGGCCTCTGCTGGCAATTCACCCAACGGAGGCTCGGCATAAGCAAAA
GATTGTGGCCCCTGTCAAGCAGACTCTGAACTTCGATTTGCTCAAACTGGCCGGCGATGTGGAGTCCA
ATCCGGGACCCGGAGACGCCCTCGTCTCC**AG**GTGAGTCCTGAGAACGTCTCTCCTGGTTTAACTCTGA
CGGTTTTTATTGTATTATTGGGGGGAAGTTCGGGTGTTGGGTCTCCTGCCAGGAGAACCCCGGAGCAG
TCTGGGTGACTCAAGAGGATGCCCTGAGGCAACCGCGACCACACAGACGAGGGGCAAGGGCTCCAGAT
GTTCCTTCCTCCTGAGCCCAACAATACTAATCTCTCTGTGGCCAGGGCCACCCTAGGCCTCTGGGGTC
CAATGCCCGACAATCCCCGGGCCCTCCCCGGACTCAGTCTGAGAGGGTCCCAGGGACGTCACGGGGCG
CCACTTCTTGCCAGGGGTCCTGGCATTGTTGTCACAATGTGACAACTAGTT

OCI-Ly19_H_HA_GFP_F2A

TTAGTGCAAAGTGTTTATCACAGCACAATTTCATAATAAGACAGCATATTTTCCAAACGCAATCATTG
CCAGCAAACTTCCACAGGGCACCGTCGTCTTATCTGGGTACAGCCTACTCCTCAAGGGTCCCACCCTA
GAGCTTGGTTTATAGTAGGAGATTTGCAAGTAGGGCCCTCCCTCTACTGATGAAAGCCAACCCAACCC
TGACCCTGCAGCTCTCAGAGAGGTGCCTTAGCCCTGGATTCCAAGGCATTTCCACTTGGTGATCAGCA
CTGAACACAGAGGACTCACCATGGTGAGCAAGGGCGAGGAGCTGTTCACCGGGGTGGTGCCCATCCTG
GTCGAGCTGGACGGCGACGTAAACGGCCACAAGTTCAGCGTGTCCGGCGAGGGCGAGGGCGATGCCAC
CTACGGCAAGCTGACCCTGAAGTTCATCTGCACCACCGGCAAGCTGCCCGTGCCCTGGCCCACCCTCG
TGACCACCTTCACCTACGGCGTGCAGTGCTTCGCCCGCTACCCCGACCACATGAAGCAGCACGACTTC
TTCAAGTCCGCCATGCCCGAAGGCTACGTCCAGGAGCGCACCATCTTCTTCAAGGACGACGGCAACTA
CAAGACCCGCGCCGAGGTGAAGTTCGAGGGCGACACCCTGGTGAACCGCATCGAGCTGAAGGGCATCG
ACTTCAAGGAGGACGGCAACATCCTGGGGCACAAGCTGGAGTACAACTACAACAGCCACAAGGTCTAT
ATCACCGCCGACAAGCAGAAGAACGGCATCAAGGTGAACTTCAAGACCCGCCACAACATCGAGGACGG
CAGCGTGCAGCTCGCCGACCACTACCAGCAGAACACCCCCATCGGCGACGGCCCCGTGCTGCTGCCCG
ACAACCACTACCTGAGCACCCAGTCCGCCCTGAGCAAAGACCCCAACGAGAAGCGCGATCACATGGTC
CTGCTGGAGTTCGTGACCGCCGCCGGGATCACTCTCGGCATGGACGAGCTGTACAAGGTGACAGAGTT
GCTGTACAGGATGAAGCGGGCCGAGACCTACTGTCCAAGGCCTCTGCTGGCAATTCACCCAACGGAGG
CTCGGCATAAGCAAAAGATTGTGGCCCCTGTCAAGCAGACTCTGAACTTCGATTTGCTCAAACTGGCC
GGCGATGTGGAGTCCAATCCGGGACCCGGAGACGCCCTCGTCTCCAGGTGAGTCCTGACAAAGTCTCA
TTTACTTTAAGTCTGAAGCCTTTTCCTGTATTTTTGGGGGGAAATACGGGTGCTGGGTCTCCTGCCTA
GAGAGCCCCGGATCAGCCTGGGAGGCTCAGGAGGACGCCCTGAGGCAACACCGACCACACGGACGAGG
GGCAAGGGCTCCAGATGTTCCTTCCTCGTGAGCCCAGTAGTGCGGGTTTCTCTGTGGCCAGGGCCACC
CTAGGCCTCTGGGGTCCAATGTCTAACAACACCCGGGCATATCAAGCTTATCGATACCGTCGACCTCG
AGGGGGGGCCCGGTACCCAGCTTTTGTTCC

U2932_H_HA_GFP_F2A

GGGTTGAACAGTACTGACCCTATTCCAAAATCTGTCCTTGATCCAGGATCACACTCATCTCTCAGACC
AGCTCCTTCAGCACATCTCTTTACCTGGAAGAAGAGGACTCTGGGCTTGGAGAGGGGAGGCCCCAAGA
AGAGAACTGAGTTCTCAAAGGGCACAGCCAGCATTCTCCTCCCAGGGTGAGCTCAAAAGACTGGCGCC
TCTCTCATCCCTTTTCACTGCTCCGTACAAACGCACCACCCCCATGCAAATCCTCACTTAGGCGCCCA
CAGGAAGCCACCACAC**ATTTCCTTAAATTCAGGTCCAACTCATAAGGGAAATGCTTTCTGAGAGCCAT**
GTATCTCATGTGCAAGAAAATGGTGAGCAAGGGCGAGGAGCTGTTCACCGGGGTGGTGCCCATCCTGG
TCGAGCTGGACGGCGACGTAAACGGCCACAAGTTCAGCGTGTCCGGCGAGGGCGAGGGCGATGCCACC
TACGGCAAGCTGACCCTGAAGTTCATCTGCACCACCGGCAAGCTGCCCGTGCCCTGGCCCACCCTCGT
GACCACCTTCACCTACGGCGTGCAGTGCTTCGCCCGCTACCCCGACCACATGAAGCAGCACGACTTCT
TCAAGTCCGCCATGCCCGAAGGCTACGTCCAGGAGCGCACCATCTTCTTCAAGGACGACGGCAACTAC
AAGACCCGCGCCGAGGTGAAGTTCGAGGGCGACACCCTGGTGAACCGCATCGAGCTGAAGGGCATCGA
CTTCAAGGAGGACGGCAACATCCTGGGGCACAAGCTGGAGTACAACTACAACAGCCACAAGGTCTATA
TCACCGCCGACAAGCAGAAGAACGGCATCAAGGTGAACTTCAAGACCCGCCACAACATCGAGGACGGC
AGCGTGCAGCTCGCCGACCACTACCAGCAGAACACCCCCATCGGCGACGGCCCCGTGCTGCTGCCCGA
CAACCACTACCTGAGCACCCAGTCCGCCCTGAGCAAAGACCCCAACGAGAAGCGCGATCACATGGTCC
TGCTGGAGTTCGTGACCGCCGCCGGGATCACTCTCGGCATGGACGAGCTGTACAAGGTGACAGAGTTG
CTGTACAGGATGAAGCGGGCCGAGACCTACTGTCCAAGGCCTCTGCTGGCAATTCACCCAACGGAGGC
TCGGCATAAGCAAAAGATTGTGGCCCCTGTCAAGCAGACTCTGAACTTCGATTTGCTCAAACTGGCCG
GCGATGTGGAGTCCAATCCGGGACCCGGAGACGCCCTCGTCTCC**AG**GTGAGTACTTACGACCTCTCTC
CTGCTTTAACTCTTAAGGATTTTGCTGCATTTTTGGGGGGAAATAAGCGTGCTGGGTCTCCTGCCAAG
AGAGCCTCGGGCTGGGTCTCCTGCCATGAGAGCCCCGGAGCAGCCTGGGGCTCAGGAGGATGCCCTGA
GGCAACAGCGGCCACACAGACGAGGGGCAAAGGCTCCAGATGTTCCTTCCTCCTGAGCCCAGCAGCAC
GGGTCTCTCTGTGGCCAGGGCCACCCTGGGCCTCTGGGGTCCAATGTCCAACAACCCCCGGGCCCTCC
CCGGGCTCAGTCTGAGAGGGTCCCAGAGACTTAACGGGGTGCCAGTTCTTGCCTGGGGTCCTCC

Homology arms for L-HVR replacement with inserted mTurqoise2 and F2A

Sequences of homology arms with inserted mTurqoise2 and F2A sequences were previously published by Havranek et al. Blood 2017.

Fragments of DNA were inserted into the pSC-B-amp/kan plasmid (Agilent Technologies) and used for ligation mediated assembly of repair template plasmids for H-HVR and L-HVR replacement.

The sequences are marked as follows:

Introns: GTTATAT
Exons in bold: **CCTTAAA**
mTurqoise2: ATGGTGA
F2A: GTGACAG
Splice donor and acceptor sites: GT AG
Silent changes to prevent re-targeting of repair template plasmid and modified genomic locus: ACTGG
BsmBI restriction cassette: GGAGACGCCCTCGTCTCC
BsmBI restriction sites: GAGACG and CGTCTC
Cas9/sgRNA sites: underlined

HBL-1_L_HA_mTruquoise2_F2A

CCATCATGCATTTAGGGAGCTGACTGGGCACAAGTTGGAGCAGAAAGAGAAAAATGAAACCACAGCCT
TCTATTTTGTTTCTAACAGACTTGTACCAAACATTCTGTGGCTCAATCTAGGTGATGGTGAGACAAGA
GGACACAGGGGTTAAATTCTGTGGCCGCAGGGGAGAAGTTCTACCCTCAGACTGAGCCAACGGCCTTT
TCTGGCCTGATCACCTGGGCATGGGCTGCTGAGAGCAGAAAGGGGAGGCAGATTGTCTCTGCAGCTGC
AAGCCCAGCACCCGCCCCAGCTGCTTTGCATGTCCCTCCCAGCCGCCCTGCAGTCCAGAGCCCATATC
AATG**CCTGGGTCAGAGCTCTGGAGAAGAGCTGCTCGGTCAGTACCCAGAGGGAACC**ATGGTGAGCAAG
GGCGAGGAGCTGTTCACCGGGGTGGTGCCCATCCTGGTCGAGCTGGACGGCGACGTAAACGGCCACAA
GTTCAGCGTGTCCGGCGAGGGCGAGGGCGATGCCACCTACGGCAAGCTGACCCTGAAGTTCATCTGCA
CCACCGGCAAGCTGCCCGTGCCCTGGCCCACCCTCGTGACCACCCTGTCCTGGGGCGTGCAGTGCTTC
GCCCGCTACCCCGACCACATGAAGCAGCACGACTTCTTCAAGTCCGCCATGCCCGAAGGCTACGTCCA
GGAGCGCACCATCTTCTTCAAGGACGACGGCAACTACAAGACCCGCGCCGAGGTGAAGTTCGAGGGCG
ACACCCTGGTGAACCGCATCGAGCTGAAGGGCATCGACTTCAAGGAGGACGGCAACATCCTGGGGCAC
AAGCTGGAGTACAACTACTTTAGCGACAACGTCTATATCACCGCCGACAAGCAGAAGAACGGCATCAA
GGCCAACTTCAAGATCCGCCACAACATCGAGGACGGCGGCGTGCAGCTCGCCGACCACTACCAGCAGA
ACACCCCCATCGGCGACGGCCCCGTGCTGCTGCCCGACAACCACTACCTGAGCACCCAGTCCAAGCTG
AGCAAAGACCCCAACGAGAAGCGCGATCACATGGTCCTGCTGGAGTTCGTGACCGCCGCCGGGATCAC
TCTCGGCATGGACGAGCTGTACAAGGTGACAGAGTTGCTGTACAGGATGAAGCGGGCCGAGACCTACT
GTCCAAGGCCTCTGCTGGCAATTCACCCAACGGAGGCTCGGCATAAGCAAAAGATTGTGGCCCCTGTC
AAGCAGACTCTGAACTTCGATTTGCTCAAACTGGCCGGCGATGTGGAGTCCAATCCGGGACCCGGAGA
CGCCCTCGTCTCC**AC**GTAAGTACATCTGTCTCAATTATTCGTGAGATTTTAGTGTCACTGAATCATTT
GTGCAAATTTTGTGATATTTTGGTTGAATAAACCTGGTGACCCAGAAGTAAATAGCAGGACACCAGAA
AATGAACTTAAAAATCTGAGCAAATAGACGAATCATTGGGTTTGAGAGGAGAATAGGATTCATGGGGG
AAATGGGGAAGAAATAGCTAGATTTTTCTCTGAACAAGCAGCCTATCTCGTATGATTGGCTTCAAGAG
AAGTTTTTGTTGAGGGGAAAGGGTGAGATCCCTCACTGTGACTCACTTTCGGCGGAGGGACCAAGGTG
GAGATCAAACATAAGTGCACTTTCCTAATGCTTTTTCTTATAAGGTTTTAAATTTGGAGCATTTTTGT
GTTTGAGATATTAGCTCAGG

OCI-Ly19_L_HA_mTruquoise2_F2A
CCCACCCTCTAAGACTTCATTAGACATTCCCTACGAATGGTTATACTCTCCTGTATACTCCCAATACA
ACTCTAAAATATATTATTCCATATAGTCCTTAGGTTTGTATTAAAGTTTGACTTTTTTCCTTCAAAAT
ATCTCTTGTCACAACAGCGGCTCTAGAGAGAAATACATTCCCTCCAGGCAAATCTATGCTGCGCTGGT
CTGACCTGGGACCCTGGGGACATTGCCCCTGTGCTGAGTTACTAAGATGAGCCAGCCCTGCAGCTGTG
CTCAGCCTGCCCCATGCCCTGCTGATTGATTTGCATGTTCCAGAGCACAGCCTCCTGCCCTGAAGACT
TTTTTAT**GGGCTGGTCGCACCCTGTGCAGGAGTCAGTCTCAGTCAGGACACAGC**ATGGTGAGCAAGGG
CGAGGAGCTGTTCACCGGGGTGGTGCCCATCCTGGTCGAGCTGGACGGCGACGTAAACGGCCACAAGT
TCAGCGTGTCCGGCGAGGGCGAGGGCGATGCCACCTACGGCAAGCTGACCCTGAAGTTCATCTGCACC
ACCGGCAAGCTGCCCGTGCCCTGGCCCACCCTCGTGACCACCCTGTCCTGGGGCGTGCAGTGCTTCGC
CCGCTACCCCGACCACATGAAGCAGCACGACTTCTTCAAGTCCGCCATGCCCGAAGGCTACGTCCAGG
AGCGCACCATCTTCTTCAAGGACGACGGCAACTACAAGACCCGCGCCGAGGTGAAGTTCGAGGGCGAC
ACCCTGGTGAACCGCATCGAGCTGAAGGGCATCGACTTCAAGGAGGACGGCAACATCCTGGGGCACAA
GCTGGAGTACAACTACTTTAGCGACAACGTCTATATCACCGCCGACAAGCAGAAGAACGGCATCAAGG
CCAACTTCAAGATCCGCCACAACATCGAGGACGGCGGCGTGCAGCTCGCCGACCACTACCAGCAGAAC
ACCCCCATCGGCGACGGCCCCGTGCTGCTGCCCGACAACCACTACCTGAGCACCCAGTCCAAGCTGAG
CAAAGACCCCAACGAGAAGCGCGATCACATGGTCCTGCTGGAGTTCGTGACCGCCGCCGGGATCACTC
TCGGCATGGACGAGCTGTACAAGGTGACAGAGTTGCTGTACAGGATGAAGCGGGCCGAGACCTACTGT
CCAAGGCCTCTGCTGGCAATTCACCCAACGGAGGCTCGGCATAAGCAAAAGATTGTGGCCCCTGTCAA
GCAGACTCTGAACTTCGATTTGCTCAAACTGGCCGGCGATGTGGAGTCCAATCCGGGACCCGGAGACG
CCCTCGTCTCC**AC**GTGAGTGGGATTTACACTTTGTTTCTTCACTTGTCTGTGTCTTTTGTTCTCTGCG
TATATGAAGTGATATATAAGGTTAGTCTAGAAGCAGTCTGTGACATCCTTCAGGGAAAAGGTTGATA
AGTCTGGAATCAAACTCGAAAATTGATTACACATTTTTTTGAGGAATAATCAACCTTCAGGCATTGG
GTGAGAATAAGTCGTCTACGTAATAATTTAGAGATGTCGTGGGGAACATAACATGTTCTGGACAGAAC
CTTGGTCAATTGTCACAAAGGGAATTTTTGCATAGGAGGCAAAGTAAGTGGAACCAACGTGTATACAC
TTTTGGCCAGGGGACCAAGG

U2932_L_HA_mTruquoise2_F2A
CCATCATGCATTTAGGGAGCTGACTGGGCACAAGTTGGAGCAGAAAGAGAAAAATGAAACCACAGCCT
TCTATTTTGTTTCTAACAGACTTGTACCAAACATTCTGTGGCTCAATCTAGGTGATGGTGAGACAAGA
GGACACAGGGGTTAAATTCTGTGGCCGCAGGGGAGAAGTTCTACCCTCAGACTGAGCCAACGGCCTTT
TCTGGCCTGATCACCTGGGCATGGGCTGCTGAGAGCAGAAAGGGGAGGCAGATTGTCTCTGCAGCTGC
AAGCCCAGCACCCGCCCCAGCTGCTTTGCATGTCCCTCCCAGCCGCCCTGCAGTCCAG**AGCCCATATC**
AATGCCTGGGTCAGAGCTCTGGAGAAGAGCTGCTCAGTCAGTACCCTGCGGGAACCATGGTGAGCAAG
GGCGAGGAGCTGTTCACCGGGGTGGTGCCCATCCTGGTCGAGCTGGACGGCGACGTAAACGGCCACAA
GTTCAGCGTGTCCGGCGAGGGCGAGGGCGATGCCACCTACGGCAAGCTGACCCTGAAGTTCATCTGCA
CCACCGGCAAGCTGCCCGTGCCCTGGCCCACCCTCGTGACCACCCTGTCCTGGGGCGTGCAGTGCTTC
GCCCGCTACCCCGACCACATGAAGCAGCACGACTTCTTCAAGTCCGCCATGCCCGAAGGCTACGTCCA
GGAGCGCACCATCTTCTTCAAGGACGACGGCAACTACAAGACCCGCGCCGAGGTGAAGTTCGAGGGCG
ACACCCTGGTGAACCGCATCGAGCTGAAGGGCATCGACTTCAAGGAGGACGGCAACATCCTGGGGCAC
AAGCTGGAGTACAACTACTTTAGCGACAACGTCTATATCACCGCCGACAAGCAGAAGAACGGCATCAA
GGCCAACTTCAAGATCCGCCACAACATCGAGGACGGCGGCGTGCAGCTCGCCGACCACTACCAGCAGA
ACACCCCCATCGGCGACGGCCCCGTGCTGCTGCCCGACAACCACTACCTGAGCACCCAGTCCAAGCTG
AGCAAAGACCCCAACGAGAAGCGCGATCACATGGTCCTGCTGGAGTTCGTGACCGCCGCCGGGATCAC
TCTCGGCATGGACGAGCTGTACAAGGTGACAGAGTTGCTGTACAGGATGAAGCGGGCCGAGACCTACT
GTCCAAGGCCTCTGCTGGCAATTCACCCAACGGAGGCTCGGCATAAGCAAAAGATTGTGGCCCCTGTC
AAGCAGACTCTGAACTTCGATTTGCTCAAACTGGCCGGCGATGTGGAGTCCAATCCGGGACCCGGAGA
CGCCCTCGTCTCC**AC**GTGAGTAGAATTTAAAGTTTGCTTACTTAGTTGTCTGTGTCTTCTGCTTCCGG
TGTCTATGAAGTGATCTATAAACTGACTCTGCAATCAGCCTCTGATATCCTTCAGGGAAAAGAAAAAG
ATAAGTCTGTAGTCAAACTCGAGAATTGATTGCACATTTTCTTTGAAGAGCAAGCAAGATTCAGTCAT
TGGGTGAGAATAACTTGTCTAAGTAATAGCTTCAGAAATGTCCTGGGGAACATAACATGTTCTGGACA
GAGCCTTGGTCAATTGTCAGAAAGGGAGTTTTGTATAGGAGGGAAGTTAAGAGGAACCATTGTGTGT
ACACTTTTGGCCAGGGGACCAAGCTGGAGATCAAACGTAAGTACTTTTTTCCACTGATTCTTCACTGT
TGCTA

Endogenous H-HVR and L-HVR fragments

Sequences of H-HVR and L-HVR were previously published by Havranek et al. Blood 2017.

Fragments of DNA were inserted into the pSC-B-amp/kan plasmid (Agilent Technologies) and used for ligation mediated assembly of repair template plasmids for H-HVR and L-HVR replacement.

The sequences are marked as follows:
Introns: GTTATAT
Exons in bold: **CCTTAAA**
F2A: GTGACAG
Splice donor and acceptor sites: GT AG
Silent changes to prevent re-targeting of repair template plasmid and modified genomic locus: ACTGG
BsmBI restriction sites: GAGACG and CGTCTC
BbsI restriction sites: GAAGAC and GTCTTC
Cas9/sgRNA sites: underlined

HBL-1_H-HVR

GGACACTGAGAAGACCTACCC**AAACACCTGTGGTTCTTCCTCCTCCTGGTGGCAGCTCCCGGAT**GTGA
GTGTCTCAGGAATGCGGATATGAAGATATGAGATGCTGCCTCTGATCCCAGGACTCACTGTGGGTTTC
TCTGTTCACAGGGGTCCTGTCCCAGGTGCAGCTACAGCAGTGGGGCGCAGGACTGTTGAAGCCTTCGG
AGACCCTGTCCCTCACTTG**CGCTGTCTATGGTGGGTCCTTCAGTGATTACTACTGGACCTGGATCCGT**
CAGTCCCCAGGAAAGGGGCTGGAGTGGATTGGGGAAATCAATCGTAGTGGAAGTACCGACTACAACCC
GTCCCTCAAGAGTCGAGTCACCATATCACTAGACACGTCCAAGAACCAATTCTCCCTGCATCTGACCT
CTGTGACCGCCGCGGACACGGCTCTATATTACTGTGCGGGGGGACAAGACTACGGTGACTATGTTAGG
GGGGGTAGTGACTACTGGGGCCAGGGAAACCTGGTCACCGTCTCCTCAGGTGGGTCTTCGACC

HBL-1_L-HVR

GGAACGTCTCCACCC**GAGACACCCGCGCAGCTTCTCTTCCTCCTGCTACTCTGGCTCCCAG**
GTGAGGGGAACATGGGATGGTTTTGCATGTCAGTGAAAACCCTCTCAAGTCCTGTTACCTGGCAACTC
TGCTCAGTCAATACAATAATTAAAGCTCTGTATAAAGCAATAATTCTGGCTCTTCTGGGAAGACAATG
GGTTTGATTTAGATTACATGGGTGACTTTTCTGTTTTATTTCCAATCTCAG**ATACCACCGGAGAAATT**
GTGTTGACGCAGTCTCCAGGCACCCTGTCTTTGTCTCCAGGGGAAAGAGCCACCCTCTCCTGCAGGGC
CAGTCAGAGTATTAGCAGCAACTACTTAGCCTGGTTCCAGCTGAAAGGTGGCCAGGCTCCCAGGCTCC
TCATCTTTGGTGCATCCAACAGGGCCACTGGCATCCCAGACAGGTTCAGTGGCAGTGGGTCTGGGACA
GACTTCACTCTCACCATCAGCAGACTGGAGCCTGAAGATTTTGCAGTGTATTACTGTCAGCAGTATGG
TAGCTCACCGATCACTTTCGGCCCTGGCACAAAGGTGGATATCAAAC**GT**GGAGACGAAACC

OCI-Ly19_H-HVR

GGATGTCGTCTCCACCC**AAGTTGGGGCTGTGCTGGGTTTTCCTTGTTGGTATTTTAGAAG**GTGATTCA
TGGAAAACTAGAGAGATTTAGTGTGTGTGGATATGAATGAGACAAACAGTGGATATGTGTGGCAGTTT
CTGATTTTGGTGTCTCTTTGTTTGCAG**GTGTCCAGTGTGAAGTTGAGTTGGTGGAGTCTGGGGGAGGG**
TTGGTACAGCCTGGGGGGTCCTTGAGACTCTCCTGTGAAGTCTCTGGATTCACCTTCAATACCTATAC
TATGAGCTGGGTCCGCCAGGCTCCAGGTAAGGGGCTGGAGTGGGTTTCAAATATTAGTAGTAGTAGTA

GTGCCATATACTATGCAGGCTCTGTGAAGGGCCGATTCATCATCTCCAGAGACAATGCCAAAAACTCA
TTATATCTGCAAATGAACAACCTGAGAGCCGAGGACACGGCTGTCTATTTCTGTGCGCGAGCGTCTTA
TGATTCGGGGACTTATTTCCACGACTACTGGGGCCAGGGAACACTCGTCACAGTCTCCTCAGGTG**GAG**
ACGACC

OCI-Ly19_L-HVR
GGAACGTCTCCACCC**GACATGAGGGTCCCCGCTCAGCTCCTGGGGCTCCTGCTACTCTGGCTCCGAG**G
TAAGGATGGAGAACACTAGGAATTTACTCAGCCAGTGTACTCAGTACTGATCGAAGCTTCAGGGAAAA
TCTCTGATAACATGATTAGTAGTAAAAATCTTTGTTTTTATGTTTTCACTTTCAG**GTGCCAGATGTGA**
CATCCAGATGACCCAGTCTCCCTCGTCCCTGTCAGCATCTGTAGGAGACAGAGTCACTATCACTTGCC
GGGCAAGTCAGAATATTAGGACCAATTTAAATTGGTATCAACAAAAACCAGGGAGGGCCCCTAAGGTC
CTGATCTATGCCGCTTCCAGTTTGCAAAGTGGAGTCCCATCAAGATTCAGTGGCAGTGGATCTGGGAC
ATATTTCACTCTCACCATTAGCAGTCTGCAGCCTGAGGATTTTGCAACTTTCTATTGTCAACAGACGT
ACAGTTCGTCGTGGACGTTCGGCCAAGGCACAAAAGTGGAAATCAGACGTGGAGACGAAACC

U2932_H-HVR
GGACGAAGACCTACCC**AAGCACCTGTGGTTCTTCCTCCTGCTGGTGGCGGCTCCCAGAT**GTGAGTGTT
TCTAGGATGCAGACATGGAGATATGGGAGACTGCCTCTGATCCCAGGGCTCACTGTGGGTTTTTCTGT
TCACAG**GGGTCCTGTCCCAGCTGCAGCTGCAGGAGTCGGGCCCAGGACTGGTGAAGCCTTCGGAGACC**
CTGTCCCTCACCTGCACTGTCTCTCGTGTCTCCATCAGCAGTAGTAATTACTACTGGGGCTGGATCCG
CCAGCCCCCAGGGAAGGGGCTGGAATGGATTGGGAGTGACTATTATGGTGGCAGTACCTCCTACAACC
CGTCCCTCAAGGGTCGAGTCATTATATCCGTAGACACGTCCAAGAACCACTTCTCCCTGAAACTGACC
TCTGTGACCGCCGCAGACACGGCTCTATATTACTGTGCGAGAGCGCTGTCTTACTATGATACTGGTGG
TTTCAGGTACTTCTTCGATTATTGGGGCCAGGGAACACTGGTCACAGTGTCCTCAGGTGGGTCTTCGA
CC

U2932_L-HVR
GGAACGTCTCCACCC**GAAACCCCAGCGCAGCTTCTCTTCCTCCTGCTACTCTGGCTCCCAG**GTGAGGG
GAACATGGGATGGTTTTGCATGTCAGTGAAAACCCTCTCAAGTCCTGTTACCTGGCAACTCTGCTCAG
TCAATTCAATAATTAAAGCTCAATATAAAGCAATAATTCTGGCTCTTCTGGGGAGACAATGGGTTTGA
TTTAGATTACATGGGTGACTTTTCTGTTTTATTTCCAATCTCAG**ATACCACCGGAGAAATTGTGTTGA**
CGCAGTCTCCAGGCACCCTGTCTTTGTCTCCAGGGGAAAGAGCCACCCTCTCCTGCAGGGCCAGTCAG
AGTGTTAGCAGCAGCTACTTAACCTGGTACCAGCAGAAACCTGGCCAGGCTCCCAGGCTCCTCATCTA
TGGTGCGTCCAACAGGGCCACTGGCATCCCAGACAGGTTCAGTGGCAGTGGGTCTGGGACAGACTTCA
CTCTCACCATCAGCAGACTGGAGCCTGAAGATTTTGCAGTGTATTACTGTCAGCAGTATCGTAGCTCA
CCTCCGACGTGGACGTTCGGCCAAGGGACAAAAGTCGAGATCAAACGTGGAGACGAAACC

Ovalbumin (OVA) recognizing HVR fragments

Fragments of DNA were inserted into the pSC-B-amp/kan plasmid (Agilent Technologies) and used for ligation mediated assembly of repair template plasmids for H-HVR and L-HVR replacement.

The sequences are marked as follows:
Introns: GTTATAT
Exons in bold: **CCTTAAA**
F2A: GTGACAG
Published sequence that used to re-create the HVR underlined **GGCTGT**
Splice donor and acceptor sites: GT AG
Changes to disrupt low GC window for better gBlock Synthesis C
BsmBI restriction sites: GAGACG and CGTCTC

OVA-H-HVR

GGAACGTCTCCACCCGGATGGAGCTGTATCATCCTCTTTTTGGTAGCAACAGCTACAGGTAAGGGGCT
CACAGTAGCAGGCTTGAGATCTGGCAATACACTTGGGTGACAATGACATCCACTCTCTCTTTCTCTCC
ATAGGTGTCCACTCCCAGGTCCAACTGCAGCAGCCTGGGGCTGTGTTGGTGAGGCCTGGGGCTTCAGT
GAAGCTGTCCTGTAAGGCTTCTGGCTACATCTTCACCAGTTACTGGATGAATTGGGTGAAACAGAGGC
CTGGACAAGGCCTTGAATGGATTGGTATGATTGATTGTTCAGACAGAAAAACTCACTACAATCAAATG
TTCAAAGACAAGGCCACATTGACTGTTGACAAGTCCTCCAATATAGCCTACATTCAGCTCATCAGTCT
GACATCTGAGGACTCTGCGGTCTATTACTGTTCAAGGGGGAGTAAATACTGGGGCCAAGGGACTCTGG
TCACTGTCTCTTCAGGTGGAGACGAAACC

OVA-L-HVR

GGAACGTCTCCACCCATGAGTCCTGCCCAGTTCCTGTTTCTGTTAGTGCTCTGGATTCGGGGTAAGGA
GTTCTGGAATGGGAGGGATGAGAATGGGGATGGAGGGTGATCTCTGGATGCCTATGTGTGCTGTTTAT
TTGTGGTGGGGCAGGTCATATCTTCTAGGATGTGAGGTTTTGTTACATCCTAATGAGATATTCCAGAT
GGAACAGTAGCTGTACTAAGATCAATATTCTGACATAGATTGGATGGAGTGGTATAGACTCTGATGTT
TAGAACCTTCAACATTTGTTTTATGACAAGATATTTGATATATCATATCTTTAAATCTGAAAAACTGC
TAGGATCTTACTTGAAAGGAATAGCATTTTCAAGTAAGATTTCAAGTAGATTTTCAAGTAGATTTCAC
AAAGGTTGCTCAGGACCTTTGCACATGATTTTCCACTATTGTATTGTAATTTCAGAAACCAACGGTGA
TGTTGTGATGACCCAGACTCCACTCACTTTGTCGGTTACCATTGGACAACCAGCCTCCATCTCTTGCA
AGTCAAGTCAGAGCCTCTTAGATAGTGATGGAAAGACATATTTGAATTGGTTGTTACAGAGGCCAGGC
CAGTCTCCAAAGCGCCTAATCTATCTGGTGTCTAAACTGGACTCTGGAGTCCCTGACAGGTTCAGTGG
CAGTGGGTCTGGAACGGATTTCACACTGAAAATCAGCAGAGTGGAGGCTGAGGATTTGGGAGTTTATT
ATTGCTGGCAAGCTACACATTTTCCTCAGACGTTCGGTGGAGGTACCAAGTTGGAAATCAAACGTGGA
GACGAAACC

Sequences of murine CD8a (mCD8) protein and truncated mCD8a, fused to 17mer-OVA peptide

mCD8A sequence

ATGGCCTCACCGTTGACCCGCTTTCTGTCGCTGAACCTGCTGCTGCTGGGTGAGTCGATTATCCTGGGGAGTGGA
GAAGCTAAGCCACAGGCACCCGAACTCCGAATCTTTCCAAAGAAAATGGACGCCGAACTTGGTCAGAAGGTGGAC
CTGGTATGTGAAGTGTTGGGGTCCGTTTCGCAAGGATGCTCTTGGCTCTTCCAGAACTCCAGCTCCAAACTCCCC
CAGCCCACCTTCGTTGTCTATATGGCTTCATCCCACAACAAGATAACGTGGGACGAGAAGCTGAATTCGTCGAAA
CTGTTTTCTGCCATGAGGGACACGAATAATAAGTACGTTCTCACCCTGAACAAGTTCAGCAAGGAAAACGAAGGC
TACTATTTCTGCTCAGTCATCAGCAACTCGGTGATGTACTTCAGTTCTGTCGTGCCAGTCCTTCAGAAAGTGAAC
TCTACTACTACCAAGCCAGTGCTGCGAACTCCCTCACCTGTGCACCCTACCGGGACATCTCAGCCCCAGAGACCA
GAAGATTGTCGGCCCCGTGGCTCAGTGAAGGGGACCGGATTGGACTTCGCCTGTGATATTTACATCTGGGCACCC
TTGGCCGGAATCTGCGTGGCCCTTCTGCTGTCCTTGATCATCACTCTCATCTGCTACCACAGGAGCCGAAAGCGT
GTTTGCAAATGTCCCAGGCCGCTAGTCAGACAGGAAGGCAAGCCCAGACCTTCAGAGAAAATTGTGTAA
Signal peptide, extracellular, transmembrane, and intracellular.

OVA 17-mer

TTCGACAAGTTGCCCGGCTTCGGAGATTCCATCGAAGCCCAGGGGGGCAAG

Translated: FDKLPGFGDSIEAQGGK

mCD8a-flag-2xOVA-17-mer

GGAAGGCCTCTGAGGCCACCATGGCCTCACCGTTGACCCGCTTTCTGTCGCTGAACCTGCTGCTGCTGGGTGA
GTCGATTATCCTGGGGAGTGGAGAAGCTAAGCCACAGGCACCCGAACTCCGAGACTATAAAGACGACGACGAC
AAAGACTATAAGGACGACGATGACAAGGATTACAAGGACGACGACGATAAGTTCGACAAGTTGCCCGGCT
TCGGAGATTCCATCGAAGCCCAGGGGGGCAAGGGAGGAGGCAGCGGTGGTGGAAGTGGATTCGACAAGTTGC
CCGGCTTCGGAGATTCCATCGAAGCCCAGGGGGGCAAGGATTGTCGGCCCCGTGGCTCAGTGAAGGGGACCGGAT
TGGACTTCGCCTGTGATATTTACATCTGGGCACCCTTGGCCGGAATCTGCGTGGCCCTTCTGCTGTCCTTGATCA
TCACTCTCATCTGCTACCACAGGAGCCGATAAGGCCTGTCAGGCCAACC

SfiI site, Signal peptide, part of extracellular domain, 3x flag, OVA-17-mer, linker, OVA-17-mer, part of extracellular domain, transmembrane domain, part of intracellular domain, STOP, SfiI site.

Translated correctly:
MASPLTRFLSLNLLLLGESIILGSGEAKPQAPELRDYKDDDDKDYKDDDDKDYKDDDDKFDKLPGFGD
SIEAQGGKGGGSGGGSGFDKLPGFGDSIEAQGGKDCRPRGSVKGTGLDFACDIYIWAPLAGICVALLL
SLIITLICYHRSR*

Sequences of wild-type and K44A-mutant DNM2

dymamin 2 WT

ACCACTTCCTACCCTCGAAAGGCCTCTGAGGCCACCatgggcaaccgcgggatggaagagct
gatcccgctggtcaacaaactgcaggacgccttcagctccatcggccagagctgccacctgg
acctgccgcagatcgctgtagtgggcggccagagcgccggcaagagctcggtgctggagaac
ttcgtgggccgggacttccttccccgcggttcaggaatcgtcacccggcggcctctcattct
gcagctcatcttctcaaaaacagaacatgccgagtttttgcactgcaagtccaaaaagttta
cagactttgatgaagtccggcaggagattgaagcagagaccgacagggtcacggggaccaac
aaaggcatctccccagtgcccatcaaccttcgagtctactcgccacacgtgttgaacttgac
cctcatcgacctcccgggtatcaccaaggtgcctgtgggcgaccagcctccagacatcgagt
accagatcaaggacatgatcctgcagttcatcagccgggagagcagcctcattctggctgtc
acgcccgccaacatggacctggccaactccgacgccctcaagctggccaaggaagtcgatcc
ccaaggcctacggaccatcggtgtcatcaccaagcttgacctgatggacgagggcaccgacg
ccagggacgtcttggagaacaagttgctcccgttgagaagaggctacattggcgtggtgaac
cgcagccagaaggatattgagggcaagaaggacatccgtgcagcactggcagctgagaggaa
gttcttcctctcccacccggcctaccggcacatggccgaccgcatgggcacgccacatctgc
agaagacgctgaatcagcaactgaccaaccacatccgggagtcgctgccggccctacgtagc
aaactacagagccagctgctgtccctggagaaggaggtggaggagtacaagaactttcggcc
cgacgaccccacccgcaaaaccaaagccctgctgcagatggtccagcagtttggggtggatt
ttgagaagaggatcgagggctcaggagatcaggtggacactctggagctctccgggggcgcc
cgaatcaatcgcatcttccacgagcggttcccatttgagctggtgaagatggagtttgacga
gaaggacttacgacgggagatcagctatgccattaagaacatccatggagtcaggaccgggc
ttttcaccccggacttggcattcgaggccattgtgaaaaagcaggtcgtcaagctgaaagag
ccctgtctgaaatgtgtcgacctggttatccaggagctaatcaatacagttaggcagtgtac
cagtaagctcagttcctacccccggttgcgagaggagacagagcgaatcgtcaccacttaca
tccgggaacgggagggggagaacgaaggaccagattcttctgctgatcgacattgagcagtcc
tacatcaacacgaaccatgaggacttcatcgggtttgccaatgcccagcagaggagcacgca
gctgaacaagaagagagccatccccaatcaggtgatccgcaggggctggctgaccatcaaca

acatcagcctgatgaaaggcggctccaaggagtactggtttgtgctgactgccgagtcactg
tcctggtacaaggatgaggaggagaaagagaagaagtacatgctgcctctggacaacctcaa
gatccgtgatgtggagaagggcttcatgtccaacaagcacgtcttcgccatcttcaacacgg
agcagagaaacgtctacaaggacctgcggcagatcgagctggcctgtgactcccaggaagac
gtggacagctggaaggcctcgttcctccgagctggcgtctaccccgagaaggaccaggcaga
aaacgaggatggggcccaggagaacaccttctccatggacccccaactggagcggcaggtgg
agaccattcgcaacctggtggactcatacgtggccatcatcaacaagtccatccgcgacctc
atgccaaagaccatcatgcacctcatgatcaacaatacgaaggccttcatccaccacgagct
gctggcctacctatactcctcggcagaccagagcagcctcatggaggagtcggctgaccagg
cacagcggcgggacgacatgctgcgcatgtaccatgccctcaaggaggcgctcaacatcatc
ggtgacatcagcaccagcactgtgtccacgcctgtacccccgcctgtcgatgacacctggct
ccagagcgccagcagccacagccccactccacagcgccgaccggtgtccagcatacaccccc
ctggccggcccccagcagtgaggggccccactccagggccccccctgattcctgttcccgtg
gggcagcagcctccttctcggcgcccccaatcccatcccggcctggaccccagagcgtgtt
tgccaacagtgacctcttcccagccccgcctcagatcccatctcggccagttcggatccccc
cagggattcccccaggagtgcccagcagaagacccccctgctgcgcccagccggcccaccatt
atccgcccagccgagccatccctgctcgacTAGGGCCTGTCAGGCCAAGCTTCCATCGATAG
ACATGATAAGATACATTGATGAGTTTGGACAAACCACAACAAGAATG

dymamin 2 K44A

ACCACTTCCTACCCTCGAAAGGCCTCTGAGGCCACCatgggcaaccgcgggatggaagagct
gatcccgctggtcaacaaactgcaggacgccttcagctccatcggccagagctgccacctgg
acctgccgcagatcgctgtagtgggcggccagagcgccggcgcgagctcggtgctggagaac
ttcgtgggccgggacttccttccccgcggttcaggaatcgtcacccggcggcctctcattct
gcagctcatcttctcaaaaacagaacatgccgagtttttgcactgcaagtccaaaaagttta
cagactttgatgaagtccggcaggagattgaagcagagaccgacagggtcacggggaccaac
aaaggcatctccccagtgcccatcaaccttcgagtctactcgccacacgtgttgaacttgac
cctcatcgacctcccgggtatcaccaaggtgcctgtgggcgaccagcctccagacatcgagt
accagatcaaggacatgatcctgcagttcatcagccgggagagcagcctcattctggctgtc
acgcccgccaacatggacctggccaactccgacgccctcaagctggccaaggaagtcgatcc
ccaaggcctacggaccatcggtgtcatcaccaagcttgacctgatggacgagggcaccgacg
ccagggacgtcttggagaacaagttgctcccgttgagaagaggctacattggcgtggtgaac
cgcagccagaaggatattgagggcaagaaggacatccgtgcagcactggcagctgagaggaa
gttcttcctctcccacccggcctaccggcacatggccgaccgcatgggcacgccacatctgc
agaagacgctgaatcagcaactgaccaaccacatccgggagtcgctgccggccctacgtagc
aaactacagagccagctgctgtccctggagaaggaggtggaggagtacaagaactttcggcc
cgacgaccccacccgcaaaaccaaagccctgctgcagatggtccagcagtttggggtggatt
ttgagaagaggatcgagggctcaggagatcaggtggacactctggagctctccgggggcgcc
cgaatcaatcgcatcttccacgagcggttcccatttgagctggtgaagatggagtttgacga
gaaggacttacgacgggagatcagctatgccattaagaacatccatggagtcaggaccgggc
ttttcaccccggacttggcattcgaggccattgtgaaaaagcaggtcgtcaagctgaaagag
ccctgtctgaaatgtgtcgacctggttatccaggagctaatcaatacagttaggcagtgtac
cagtaagctcagttcctacccccggttgcgagaggagacagagcgaatcgtcaccacttaca
tccgggaacgggagggagaacgaaggaccagattcttctgctgatcgacattgagcagtcc
tacatcaacacgaaccatgaggacttcatcgggtttgccaatgcccagcagaggagcacgca
gctgaacaagaagagagccatccccaatcaggtgatccgcaggggctggctgaccatcaaca

acatcagcctgatgaaaggcggctccaaggagtactggtttgtgctgactgccgagtcactg
tcctggtacaaggatgaggaggagaaagagaagaagtacatgctgcctctggacaacctcaa
gatccgtgatgtggagaagggcttcatgtccaacaagcacgtcttcgccatcttcaacacgg
agcagagaaacgtctacaaggacctgcggcagatcgagctggcctgtgactcccaggaagac
gtggacagctggaaggcctcgttcctccgagctggcgtctaccccgagaaggaccaggcaga
aaacgaggatggggcccaggagaacaccttctccatggacccccaactggagcggcaggtgg
agaccattcgcaacctggtggactcatacgtggccatcatcaacaagtccatccgcgacctc
atgccaaagaccatcatgcacctcatgatcaacaatacgaaggccttcatccaccacgagct
gctggcctacctatactcctcggcagaccagagcagcctcatggaggagtcggctgaccagg
cacagcggcgggacgacatgctgcgcatgtaccatgccctcaaggaggcgctcaacatcatc
ggtgacatcagcaccagcactgtgtccacgcctgtacccccgcctgtcgatgacacctggct
ccagagcgccagcagccacagccccactccacagcgccgaccggtgtccagcatacaccccc
ctggccggcccccagcagtgaggggccccactccagggccccccctgattcctgttcccgtg
gggcagcagcctccttctcggcgcccccaatcccatcccggcctggaccccagagcgtgtt
tgccaacagtgacctcttcccagccccgcctcagatcccatctcggccagttcggatccccc
cagggattcccccaggagtgcccagcagaagacccccctgctgcgcccagccggcccaccatt
atccgcccagccgagccatccctgctcgacTAGGGCCTGTCAGGCCAAGCTTCCATCGATAG
ACATGATAAGATACATTGATGAGTTTGGACAAACCACAACAAGAATG

START, STOP, Mutated codon

PCR primers for insertion via in-fusion reaction into Sleeping Beauty pSB-tet-Bla plasmid :

Dynamin_01_F ACCCTCGAAAGGCCTCTGAGGCCACCatgggcaaccgcgggatg
Dynamin_01_R ATGGAAGCTTGGCCTGACAGGCCCTAGTCGAGCAGGGATGGCTCGG

Supplemental references:

1. Cong L, Ran FA, Cox D, Lin S, Barretto R, Habib N, et al. Multiplex Genome Engineering Using CRISPR/Cas Systems. Science. 2013; 339:819–23.

2. Dougan SK, Ogata S, Hu CCA, Grotenbreg GM, Guillen E, Jaenisch R, et al. IgG1+ ovalbumin-specific B-cell transnuclear mice show class switch recombination in rare allelically included B cells. Proc Natl Acad Sci U S A. 2012; 109:13739–44.

3. Havranek O, Xu J, Köhrer S, Wang Z, Becker L, Comer JM, et al. Tonic B-cell receptor signaling in diffuse large B-cell lymphoma. Blood. 2017; 130:995–1006.

4. Phelan JD, Young RM, Webster DE, Roulland S, Wright GW, Kasbekar M, et al. A multiprotein supercomplex controlling oncogenic signalling in lymphoma. Nature. 2018; 560:387–91.

5. Juszczynski P, Chen L, O'Donnell E, Polo JM, Ranuncolo SM, Dalla-Favera R, et al. BCL6 modulates tonic BCR signaling in diffuse large B-cell lymphomas by repressing the SYK phosphatase, PTPROt. Blood. 2009;114:5315-21.
