# Supplementary figures and images for: Inhibition of autoantigen-induced B-cell receptor (BCR) internalization as a therapeutic strategy in diffuse large B cell lymphoma (DLBCL)
Source: Cell Death Dis. 2026 Feb 11;17(1):216. doi: 10.1038/s41419-026-08446-1 (PMC12921016; doi:10.1038/s41419-026-08446-1)

Fig. 2 A

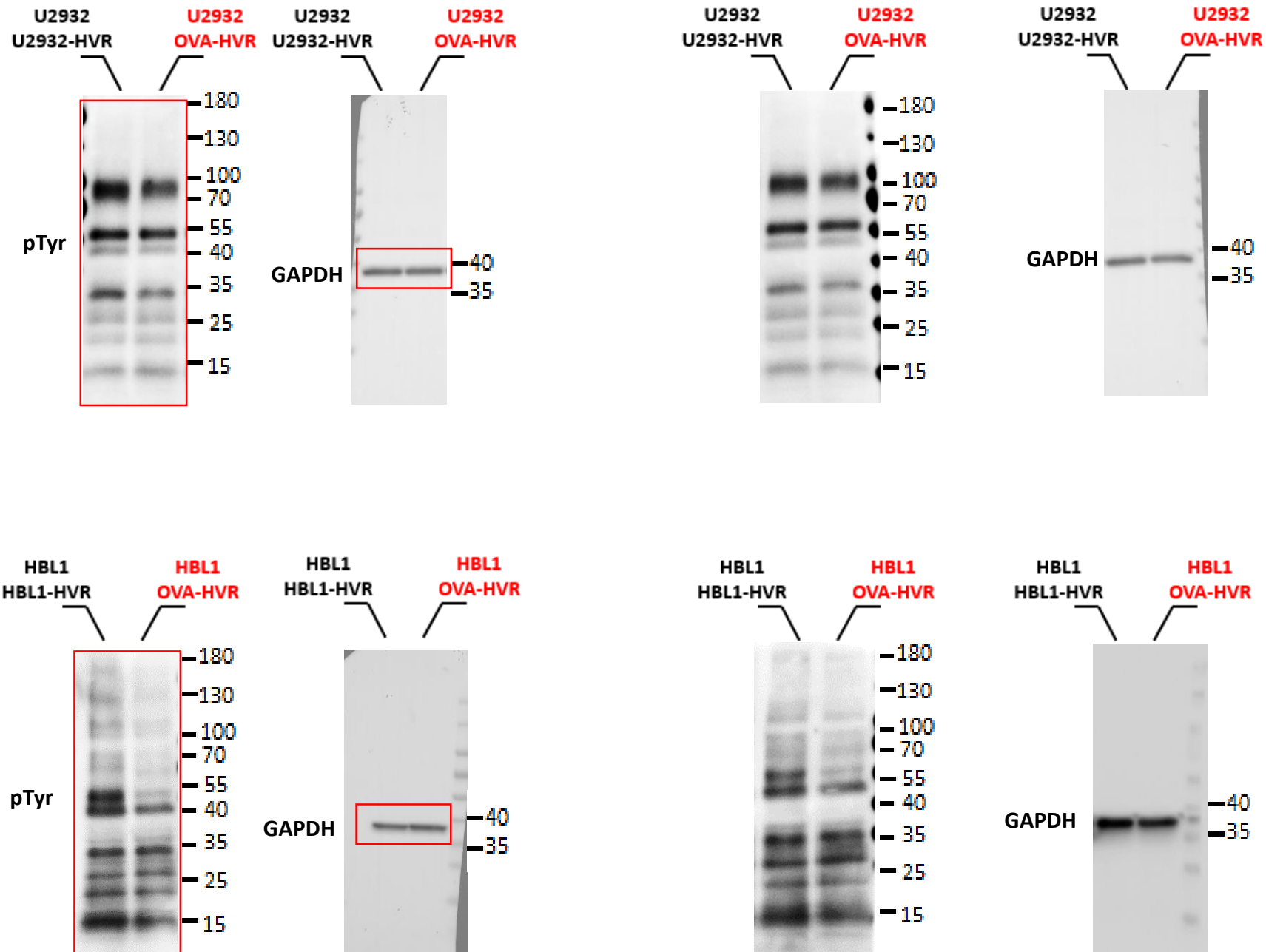

Fig. 2B

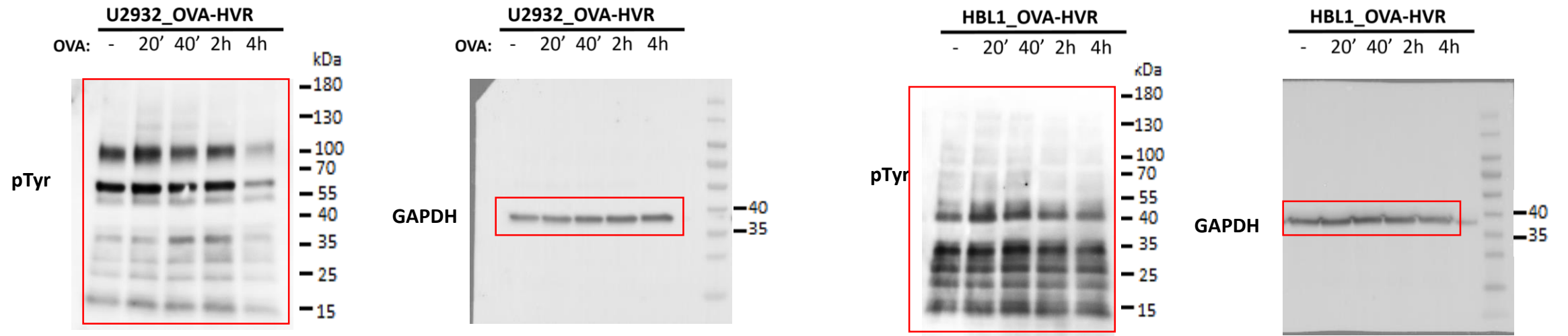

Fig. 3B

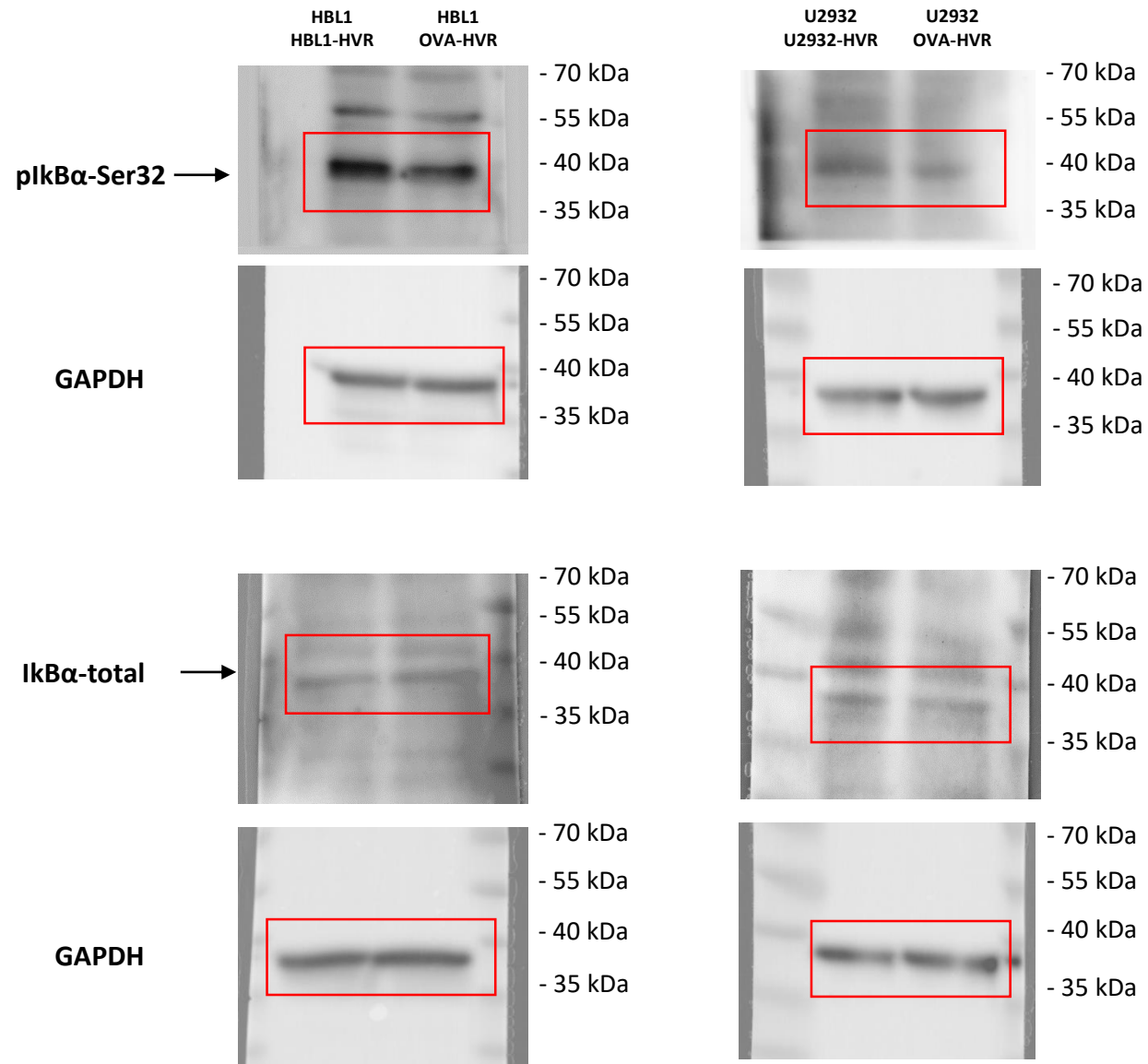

Fig. 5A

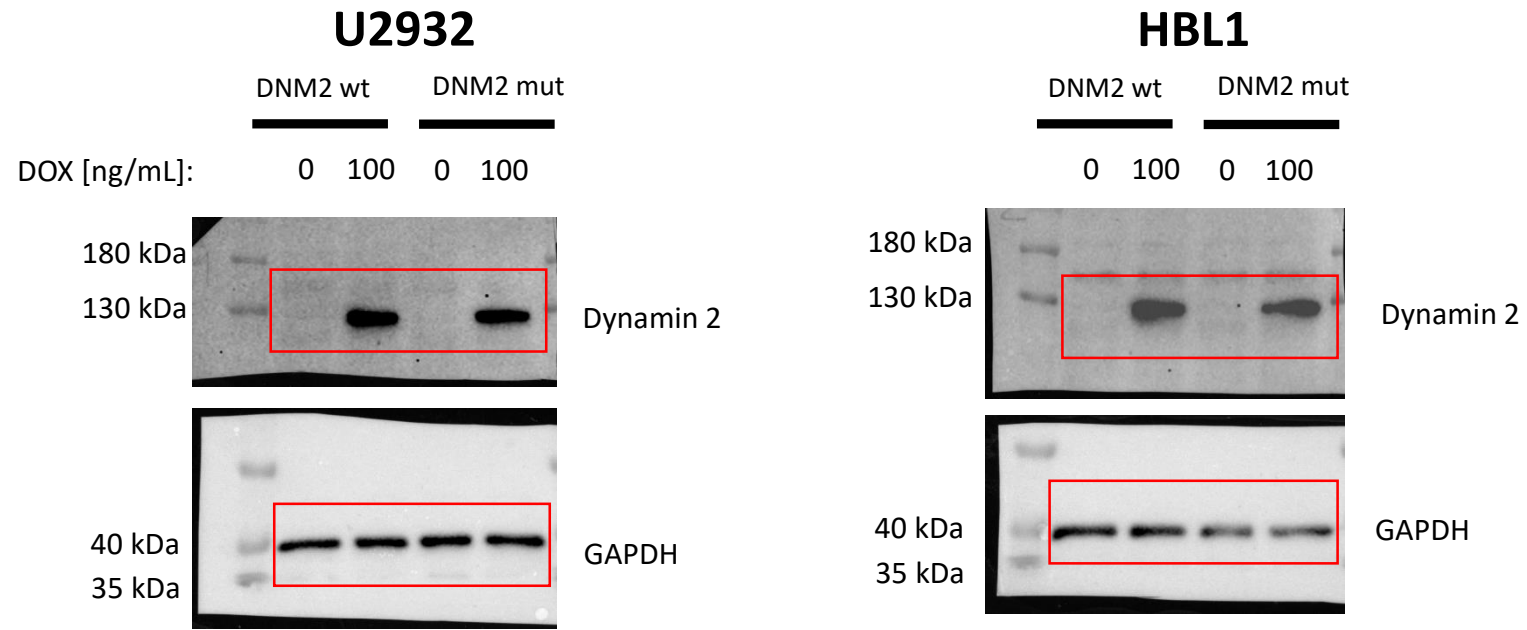

Fig. 6D

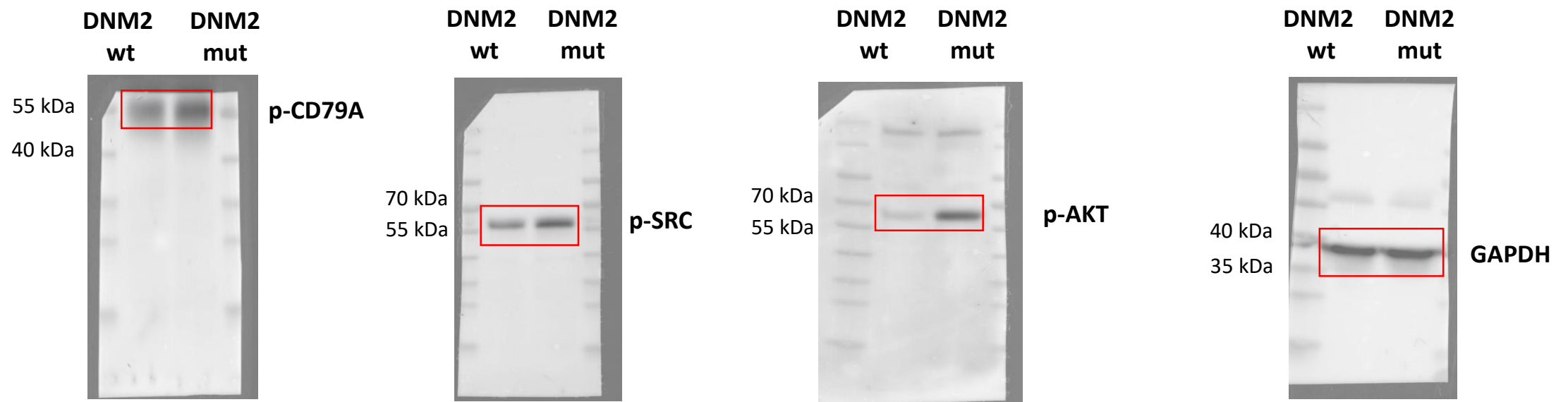

# Supplementary Figure 6

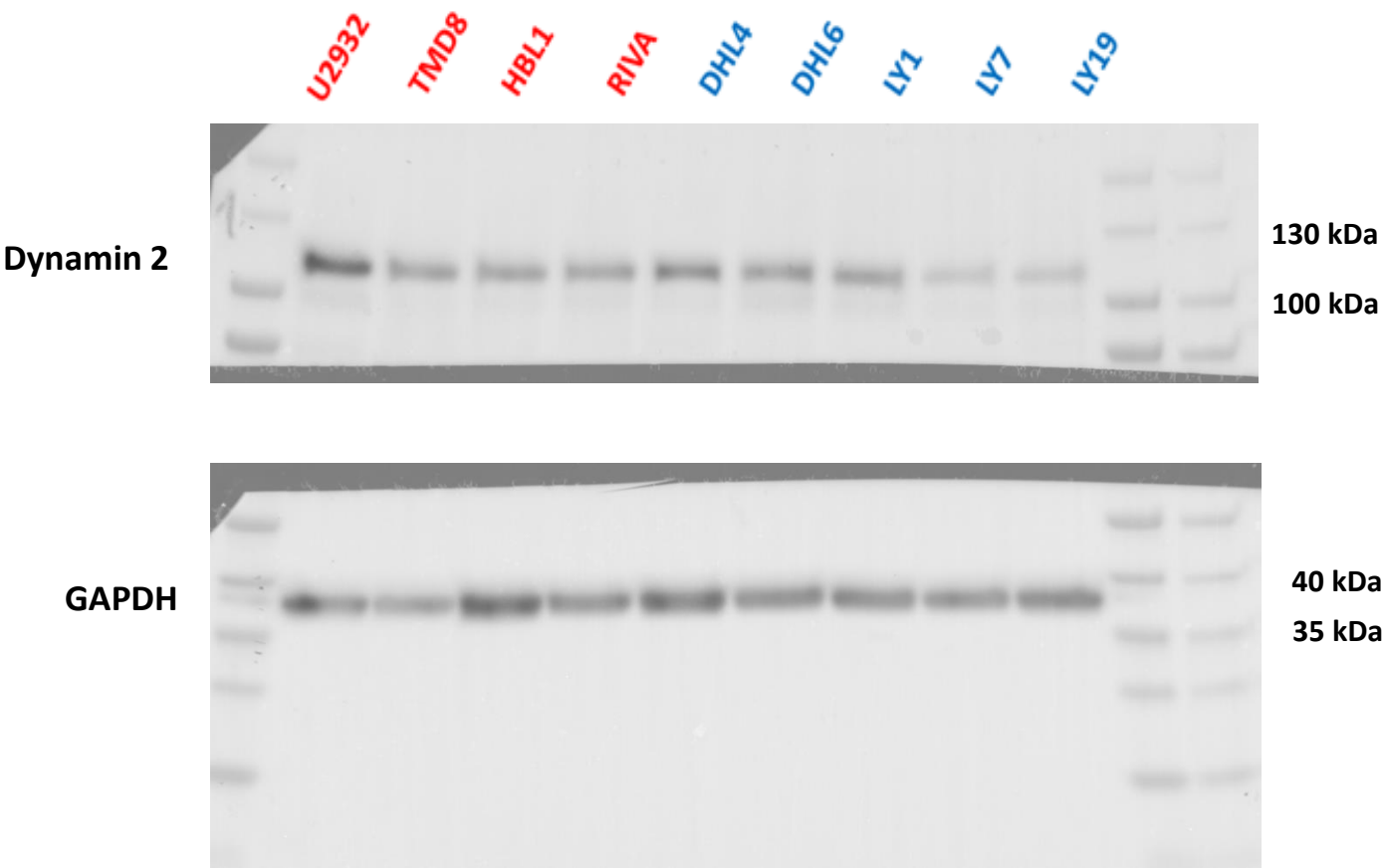

Supplement: Supplementary file 4 — Supplementary File 3 [file 41419_2026_8446_MOESM4_ESM.pdf]
